# Coherent transient exciton transport in disordered polaritonic wires

**DOI:** 10.1515/nanoph-2023-0797

**Published:** 2024-02-21

**Authors:** Gustavo J. R. Aroeira, Kyle T. Kairys, Raphael F. Ribeiro

**Affiliations:** Department of Chemistry and Cherry Emerson Center for Scientific Computation, 1371 Emory University, Atlanta, GA, USA

**Keywords:** polaritons, exciton transport, energetic disorder

## Abstract

Excitation energy transport can be significantly enhanced by strong light–matter interactions. In the present work, we explore intriguing features of coherent transient exciton wave packet dynamics on a lossless disordered polaritonic wire. Our main results can be understood in terms of the effective exciton group velocity, a new quantity we obtain from the polariton dispersion. Under weak and moderate disorder, we find that the early wave packet spread velocity is controlled by the overlap of the initial exciton momentum distribution and its effective group velocity. Conversely, when disorder is stronger, the initial state is nearly irrelevant, and red-shifted cavities support excitons with greater mobility. Our findings provide guiding principles for optimizing ultrafast coherent exciton transport based on the magnitude of disorder and the polariton dispersion. The presented perspectives may be valuable for understanding and designing new polaritonic platforms for enhanced exciton energy transport.

## 1 Introduction

The strong light–matter interaction regime is achieved when the coupling strength between light and matter overcomes dephasing and dissipative phenomena acting on each subsystem. This can be accomplished, for example, with a molecular ensemble with a narrow linewidth bright transition near resonance with an optical microcavity composed of two parallel mirrors with high-reflectivity [1], [2], [3]. In this scenario, the field confinement and low mode volumes allow light and matter to exchange energy (quasi)reversibly. Several recent studies have shown that strong light–matter coupling can be harnessed to control energy [4], [5], [6], [7], [8], [9], [10], [11], [12], [13], [14], [15], [16], [17], [18] and charge [19], [20], [21], [22], [23] transport in disordered materials. These effects are attributed to the formation of polaritons, i.e., hybrid light–matter states with intermediate properties between purely material or photonic. For example, polariton delocalization [24], [25], [26], [27] is often invoked to explain the properties of energy transport in the strong coupling regime [3].

Unlike bare excitons, which tend to show weak delocalization and inefficient energy transfer in disordered media, polaritons show much greater diversity in wave function delocalization [24], [28], [29], [30], [31] and transport phenomena [9], [16], [18]. For instance, polariton transport imaging has revealed ultrafast ballistic propagation in perovskite microcavities [16] and surface-bound polaritons [11], [18], with spread velocities spanning several orders of magnitude. The effects of disorder on polariton transport have also received significant attention as the potential source of the slower-than-expected polariton wave packet propagation reported by several groups [15], [16], [18]. Indeed, theoretical investigations suggest that dynamic and static disorder inhibit polariton wave packet propagation by effectively reducing the propagation velocity [16], [32], [33]. Interestingly, recent theoretical investigations of dipolar exciton propagation in finite one-dimensional systems suggest that under strong disorder, a disorder-enhanced transport regime emerges where coherent exciton propagation benefits from an increase in the static fluctuations of matter excitation energies [31], [33], [34].

Our recent work on coherent transport in polaritonic wires [33] thoroughly examined the requirements for convergence of exciton transport simulations with respect to model parameters. We showed that multiple (on and off-resonant) electromagnetic mode [33] played a key role in the exciton dynamics, and demonstrated the potential to control transient ballistic and diffusive exciton transport and Anderson localization under strong light–matter coupling. Here, we focus on the transient early dynamics of exciton wave packets propagating on a lossless polaritonic wire. In particular, we present numerical simulations and a detailed theoretical analysis of coherent polariton-mediated exciton transport in the ballistic regime. Our results and mathematical analysis reveal several surprising aspects of polariton-assisted coherent exciton transport, including a striking difference between the effect of disorder on ultrafast coherent exciton propagation in free space [35] and in a polaritonic medium. Furthermore, we show that an effective exciton group velocity may be defined that allows a qualitative understanding of our numerical simulations even in a moderately disordered scenario.

This article is organized as follows: in Section 2, we describe the theory and method employed in this work. Section 3 contains our main numerical results and theoretical analysis, while Section 4 provides conclusions and a summary of this work.

## 2 Theory and computation

**Figure 1:**
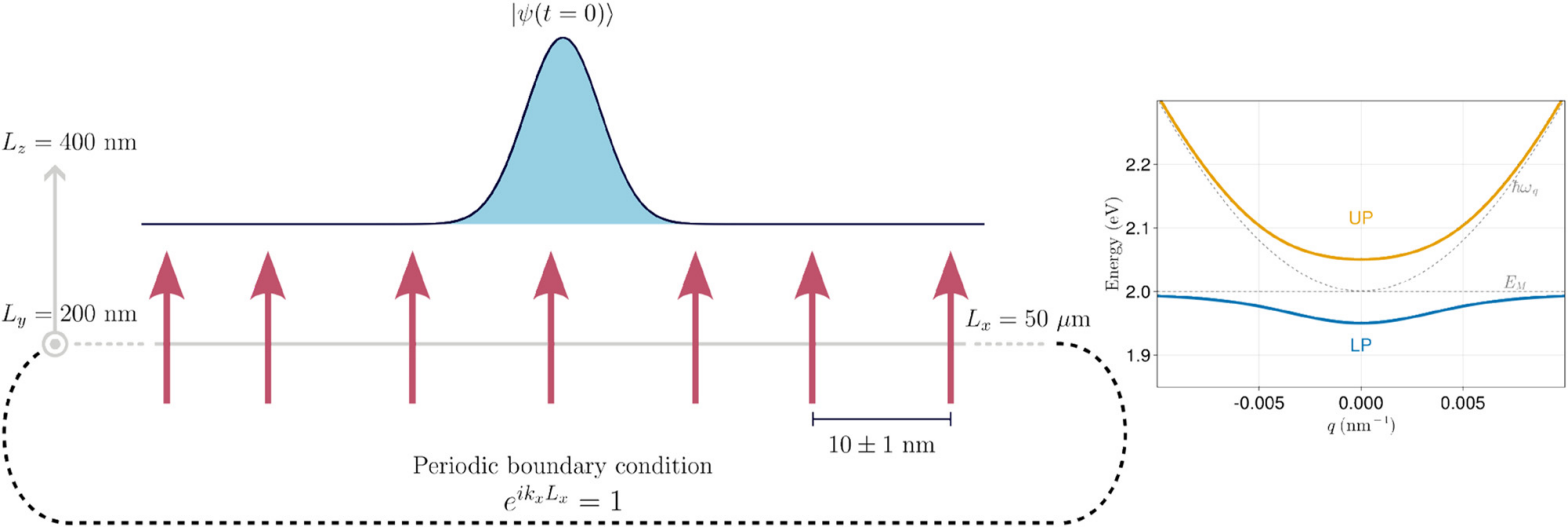
Illustration of the polariton wire model. Dipoles representing matter are noninteracting two-level systems aligned with the *z*-axis. Distances between sites are sampled from a normal distribution with an average and standard deviation of 10 and 1 nm, respectively. Likewise, excitation energies for each dipole are sampled from a normal distribution using an average of *E*_*M*_ and standard deviation of *σ*_*M*_. These parameters control the detuning and static disorder of the system. Radiation states inside the cavity are constructed using 1001 modes of the lowest energy band, with a minimum photon energy of 2.00 eV. On the right side, the dispersion for an ordered system (*σ*_*M*_ = 0) is shown. Continuous lines correspond to UP (orange) and LP (blue) branches, and dashed lines represent the bare matter (*E*_*M*_) and empty cavity (*ℏω*_*q*_) dispersions.

The polaritonic wire model employed here consists of a linear chain of dipoles representing matter (e.g., atoms, quantum wells, or molecules with negligible vibronic coupling) coupled to photon modes of a lossless cuboid optical microcavity of lengths *L*_*x*_, *L*_*y*_, and *L*_*z*_ as depicted in Figure 1. Each dipole is a two-level system with excitation energy given by ${\hat{H}}_{M}|n;0〉=E_{n}|n;0〉$ , where |*n*; 0⟩ represents a state where the *n*th dipole is in its excited state, while all other dipoles and cavity modes are in their ground states. The excitation energy of each dipole *E*_*n*_ is sampled from a normal distribution with average *E*_*M*_ and standard deviation *σ*_*M*_. Different detuning and static disorder strengths are accessed by adjusting these parameters. The spatial distribution of dipoles is also sampled from a normal distribution, but in this case, the average and standard deviation are fixed at 10 and 1 nm, respectively. The large intersite separation allows us to disregard direct interaction between these dipoles since direct energy transfer via FRET would occur at a much larger times scale than probed here. Furthermore, we also impose the same orientation for all dipoles, such that they can only interact with transverse electric (TE) polarized photon modes, and we need not consider the transverse magnetic polarization.

Imposing vanishing electric field along the *y* and *z* directions and periodic boundary conditions along the long-axis *x* implies

(1) $$k=\left(\frac{2\pi m_{x}}{L_{x}},\;\frac{\pi n_{y}}{L_{y}},\;\frac{\pi n_{z}}{L_{z}}\right),$$

where $m_{x}\in Z$ and $n_{y},n_{z}\in N_{>0}$ . Throughout this work, we employ a geometry where *L*_*x*_ = 50 μm, *L*_*y*_ = 0.2 μm, and *L*_*z*_ = 0.4 μm, such that the energy gap between adjacent bands is large enough ( >0.5 eV) that we restrict our analysis to the lowest energy band (*n*_*y*_ = 1 and *n*_*x*_ = 1). By defining $q=k_{x}=\frac{2\pi m_{x}}{L_{x}}$ and $q_{0}=\sqrt{k_{x}^{2}+k_{y}^{2}}=\sqrt{{\left(\frac{\pi }{L_{x}}\right)}^{2}+{\left(\frac{\pi }{L_{y}}\right)}^{2}}$ , it follows we can uniquely identify each photon mode using its value of *q*. The energy of each mode is

(2) $$\hbar \omega_{q}=\frac{\hbar c}{\sqrt{\epsilon }}\sqrt{q^{2}+q_{0}^{2}},$$

where *ℏ* is the reduced Planck constant, *c* is the speed of light, and *ϵ* is the relative permittivity of the intracavity medium. We use *ϵ* = 3 as a suitable parameter for organic microcavities. From Equation (2), the minimum photon energy supported in the cavity is $\hbar cq_{0}/\sqrt{\epsilon }=2.00$ eV.

The noninteracting part of the light–matter Hamiltonian is

(3) $${\hat{H}}_{0}=\underset{n}{\sum }E_{n}{\hat{b}}_{n}^{†}{\hat{b}}_{n}+\underset{q}{\sum }\hbar \omega_{q}{\hat{a}}_{q}^{†}{\hat{a}}_{q},$$

where ${\hat{b}}_{n}^{†}$ and ${\hat{a}}_{n}^{†}$ are ladder operators creating a dipolar excitation at the *n*th site and a photon in the *q* mode, respectively. We employ the Coulomb gauge in the rotating wave approximation while neglecting the diamagnetic contribution. It follows the interacting part of the Hamiltonian can be expressed as

(4) $$\begin{array}{ll} {\hat{H}}_{\text{int}} & =\overset{N_{M}}{\underset{n=1}{\sum }}\underset{q}{\sum }\frac{-i\Omega_{R}}{2}\sqrt{\frac{E_{n}}{N_{M}\hbar \omega_{q}}}\\ & \;\times \left({\text{e}}^{\text{i}qx_{n}}{\hat{b}}_{n}^{†}{\hat{a}}_{q}-{\text{e}}^{-\text{i}qx_{n}}{\hat{a}}_{q}^{†}{\hat{b}}_{n}\right), \end{array}$$

where *N*_*M*_ is the total number of sites and *x*_*n*_ is the position of the *n*th dipole along *x*. The parameter Ω_*R*_ (Rabi splitting) is related to the transition dipole moment of each molecule $\left(\vec{\mu_{d}}\right)$ by

(5) $$\Omega_{R}=\vec{\mu_{d}}\cdot \hat{z}\sqrt{\frac{\hbar \omega_{0}N_{M}}{2\epsilon L_{x}L_{y}L_{z}}}.$$

To make our study computationally tractable, we truncate the model in the number of molecules and photon modes. Following the thorough analysis in our previous work [33], we set *N*_*M*_ = 5000 to minimize finite-size effects in the sub-picosecond region. Similarly, we include 1001 cavity modes (−500 ≤ *m*_*x*_ ≤ 500), which span an energy range of 7.43 eV, well above the necessary for convergent results.

In all simulations presented here, the initial state is a Gaussian exciton wave packet with zero photonic content. This initial state can be represented in the uncoupled basis as

(6) $$\left|\psi \left(0\right)\right\rangle =\frac{1}{Z}\underset{n}{\sum }\exp\left[-\frac{{\left(x_{n}-\frac{1}{2}L_{x}\right)}^{2}}{4\sigma_{x}^{2}}+i{\bar{q}}_{0}x_{n}\right]\left|n;0\right\rangle ,$$

where *Z* is a normalization constant, *σ*_*x*_ is the initial spread of the wave packet, and ${\bar{q}}_{0}$ is the average exciton momentum along *x*. From now on, we set ${\bar{q}}_{0}=0$ unless otherwise noted. Note that *σ*_*x*_ is the standard deviation of the probability distribution *P*(*n*) = |⟨*n*; 0|*ψ*(0)⟩|^2^. The reciprocal space distribution *P*(*q*), obtained from the Fourier change of basis in (6), has standard deviation *σ*_*q*_ related to *σ*_*x*_ via

(7) $$\sigma_{x}\sigma_{q}=\frac{1}{2}.$$

We note that it may be possible to experimentally prepare exciton wave packets similar to those examined by us with surface plasmon–exciton polariton systems. In these, the molecular ensemble can be directly excited, and the incidence angle and excitation spot size may be used to control the resulting initial state (e.g., as done in ref. [36]).

In all simulations, the Fock space is truncated to include only states with one excited dipole and no photons $\left(\left|n;0\right\rangle \right)$ or one photon and no dipolar excitations $\left(\left|0;q\right\rangle \right)$ . The dynamics generated by $\hat{H}={\hat{H}}_{0}+{\hat{H}}_{\text{int}}$ and the initial state $\left|\psi \left(0\right)\right\rangle$ is in fact constrained to the single excitation subspace. Thus, our results are relevant to coherent dipolar dynamics when nonlinearities can be ignored (e.g., due to a small density of excitons). The time-evolved wave packet is directly obtained from $\left|\psi \left(t\right)\right\rangle ={\text{e}}^{-\text{i}\hat{H}t/\hbar }\left|\psi \left(0\right)\right\rangle$ using the eigenvalues and eigenvectors of $\hat{H}$ within the one-excitation manifold $\left(\left|n;0\right\rangle ⊕\left|0;q\right\rangle \right)$ .

Our computational study follows the transient evolution of exciton wave packets starting from the well-localized purely excitonic state in Equation (6). As a metric for the exciton spread, we compute the root mean square displacement of the dipolar component of the wave packet, defined here as

(8) $$\text{RMSD}\left(t\right)={\left[\frac{1}{P_{M}\left(t\right)}\overset{N_{M}}{\underset{n=1}{\sum }}|\left\langle n;0|\psi \left(t\right)\right\rangle {|}^{2}{\left(x_{n}-x_{0}\right)}^{2}\right]}^{1/2},$$

(9) $$P_{M}\left(t\right)=\overset{N_{M}}{\underset{n}{\sum }}|\left\langle n;0|\psi \left(t\right)\right\rangle {|}^{2}.$$

where the renormalization factor *P*_*M*_(*t*) is the time-dependent probability of finding any excited dipole and *x*_0_ is the average exciton position at *t* = 0 (which in this work is always $\frac{L_{x}}{2}=25$ μm). As an alternative and complementary metric of exciton mobility, we define the (matter normalized) migration probability *χ*(*t*), which measures the conditional probability of finding an excited dipole outside the region where the wave packet was initially localized. By choosing symmetric boundaries *n*_min_ ≤ *n* ≤ *n*_max_ such that there is at least 99 % chance $\left(\frac{L_{x}}{2}-3\sigma_{x}<x<\frac{L_{x}}{2}+3\sigma_{x}\right)$ that the exciton at time *t* = 0 lies within this region, we compute the migration probability as

(10) $$\chi \left(t\right)=1-\frac{1}{P_{M}\left(t\right)}\overset{n_{\max}}{\underset{n=n_{\min}}{\sum }}|\left\langle n;0|\psi \left(t\right)\right\rangle {|}^{2}.$$

All results including disorder are averages from 100 realizations. The code used in all simulations is available in our prototype package PolaritonicSystems.jl [37]. Random variables were generated using the Distributions.jl package [38], and the Makie.jl plotting ecosystem [39] was used for data visualization.

## 3 Results and discussion

### 3.1 Polariton-mediated exciton wave packet propagation

**Figure 2:**
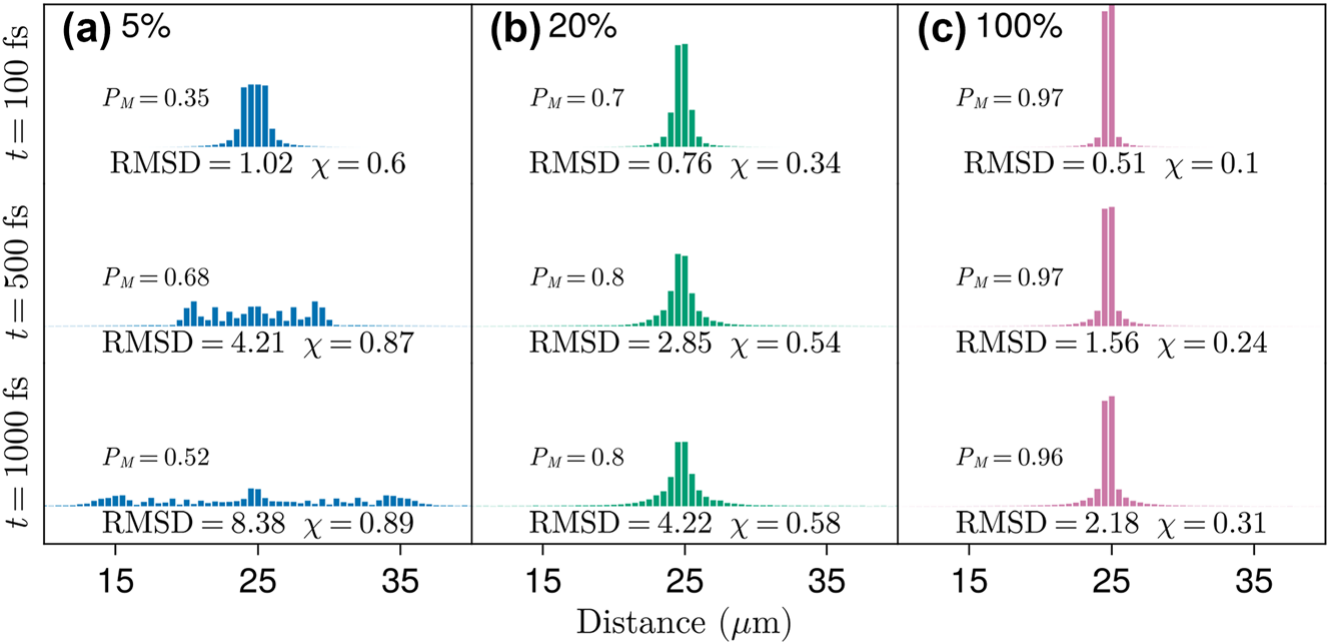
Average exciton wave packet profiles at different time delays and relative disorder strength (*σ*_*M*_/Ω_*R*_) of 5 %, 20 %, and 100 % for (a), (b) and (c), respectively. Probabilities are grouped in bins containing 50 dipoles spanning 0.5 μm. *P*_*M*_, RMSD, and *χ* are defined in Equations (8)–(10), respectively. In all cases, Ω_*R*_ = 0.1 eV and *σ*_*x*_ = 120 nm.

Selected wave packet snapshots are given in Figure 2, along with the corresponding time-dependent exciton RMSD, *P*_*M*_, and migration probability *χ*. The main effect of static disorder can be observed in these examples. From Figure 2a–c, as disorder is increased, the wave packet mobility is significantly reduced, and its spread is strongly suppressed. Simultaneously, we find the photonic content and its time-dependent fluctuations monotonically decrease and become small under strong disorder (e.g., *P*_*M*_(*t*) is relatively stable around 0.97 when *σ*_*M*_/Ω_*R*_ = 100 %). In contrast, photon content fluctuations are large under weak disorder (*σ*_*M*_/Ω_*R*_ → 0). The effect of disorder on photon content fluctuations may be directly understood from Rabi oscillations, which occur unperturbed at weak disorder while being strongly damped as *σ*_*M*_ approaches Ω_*R*_ [33]. These observations illustrate how the oscillatory energy exchange between radiation and matter leads to enhanced coherent exciton transport. Since we are examining very large values of disorder (*σ*_*M*_/Ω_*R*_ ≥ 100 %), we include in our SI (Section 8) an analysis of how the signatures of strong light–matter coupling change under increasingly stronger static disorder.

**Figure 3:**
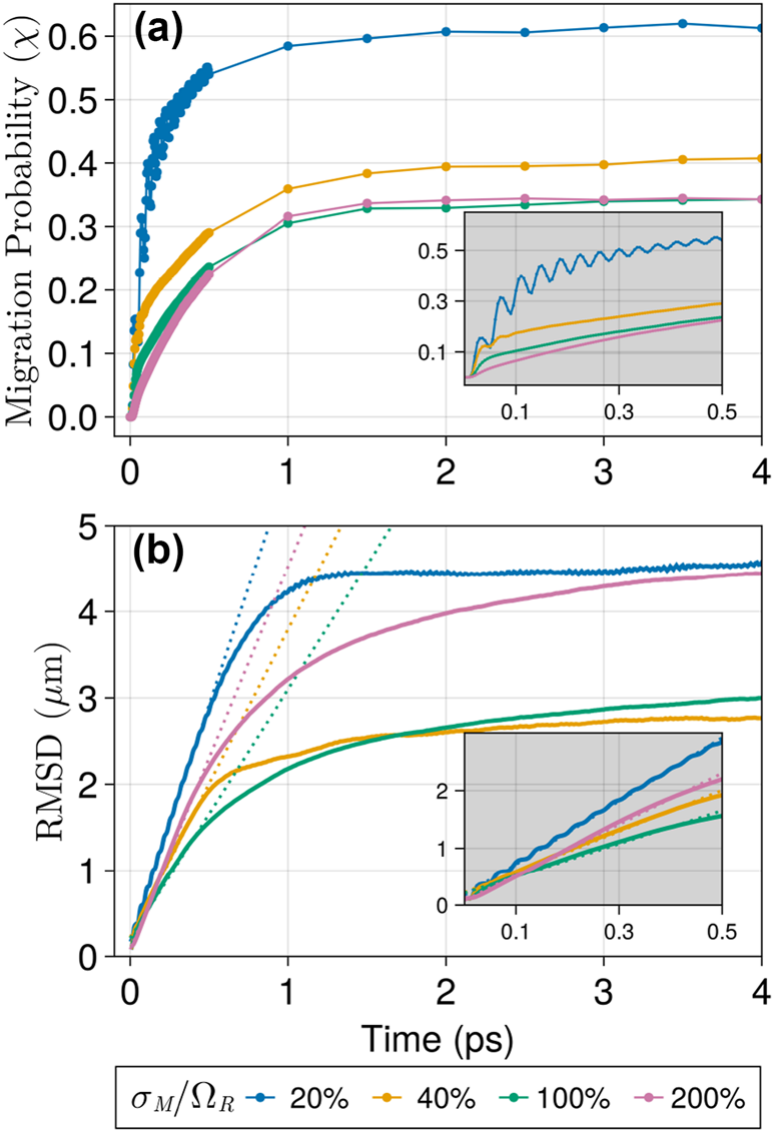
Propagation of exciton wave packets at different disorder strengths measured by (a) migration probability (Equation (10)) and (b) RMSD (Equation (8)). The dotted lines in (b) are linear fits of the early propagation (<500 fs) from which slopes are used to measure the initial ballistic velocity (*v*_0_). Insets show a zoomed-in view into the sub 500-fs region. In all cases, Ω_*R*_ = 0.1 eV and *σ*_*x*_ = 120 nm.

In Figure 3, the average exciton migration probability (Equation (10)) and RMSD are shown for excitons propagating over 4 ps under different values of relative disorder strength (*σ*_*M*_/Ω_*R*_). The migration probability, seen in Figure 3a, increases rapidly before achieving a steady state (d*χ*(*t*)/d*t* ≈ 0) around 2 ps irrespective of the disorder strength. Conversely, disorder plays a crucial role in the sub-300 fs phase of the dynamics, where we find from the inset that in all considered cases, an increase in *σ*_*M*_ leads to a slower initial exciton migration. From the behavior of *χ*(*t*) at large *t*, we find the steady-state exciton migration probability at weak disorder is largely suppressed when *σ*_*M*_ is increased. Nevertheless, the strongly disordered cases (*σ*_*M*_/Ω_*R*_ ≥ 1) suggest that beyond a particular disorder strength, *χ*(*t* ≫ 0) becomes approximately independent of *σ*_*M*_. Figures S6 and 7 provide *χ*(*t*) for Ω_*R*_ = 0.2 eV and Ω_*R*_ = 0.3 eV under different levels of disorder and verify the disorder effects on *χ*(*t*) described above are in fact generic.

In the SI Section 2, we show the initial growth of *χ*(*t*) can be approximated in the weak and strong disorder limits by analyzing the disorder-averaged properties of |⟨0; *n*|*ψ*(*t*)⟩|^2^. This leads to the conclusion that ∂*χ*(*t*)/∂*t* → 0 as *t* → 0^+^, so that the quantity *G* = (1/2)∂^2^ *χ*(*t*)/∂*t* ^2^, *t* → 0^+^ controls the early growth of *χ*(*t*). In the weak and strong disorder limits, *G* satisfies, respectively

(11) $$G^{W}\approx \frac{1}{2N_{I}}\underset{A,B\neq A}{\sum }\underset{n\in I}{\sum }|A_{n}{|}^{2}|B_{n}{|}^{2}{\left(\omega_{A}-\omega_{B}\right)}^{2},$$

(12) $$G^{S}\approx \frac{1}{2}\underset{A,B\neq A}{\sum }\underset{n\in I}{\sum }\bar{|A_{n}{|}^{2}|B_{n}{|}^{2}}|c_{n}{|}^{2}{\left(\omega_{A}-\omega_{B}\right)}^{2},$$

where *A* and *B* are eigenstates, *A*_*n*_ and *B*_*n*_ the probability amplitude to detect an exciton at the *n*th dipole when the system is in the *A* and *B* eigenstates, respectively, $I=\left[n_{\min},n_{\max}\right]$ (see Equation (10) and accompanying description), $N_{I}$ is the number of sites in I , *c*_*n*_ is the *n*th exciton amplitude in the initial wave packet, and $\bar{f}$ is the disorder average of quantity *f*. Both approximations to *G*, in the weakly and strongly disordered regimes suggest ultrafast increase in the exciton migration probability depends on the existence of eigenstates with large energy differences and significant contributions from the dipoles comprising the initial wave packet.

From Equation (12), we infer (i) the steep increase of *χ*(*t*) at early times is enhanced by raising Ω_*R*_ at fixed energetic disorder (as the energy difference between polariton modes formed from near-resonant photon and excitons increase with Ω_*R*_) and (ii) increasing disorder with fixed Ω_*R*_ leads to initial slower growth of $\chi \left(t\to 0^{+}\right)$ , due to the greater tendency of localization of the *n*th exciton into strongly localized eigenmodes. Importantly, while the summand of Equation (11) has the same form as that of Equation (12), the former has several more non-negligible contributions than the latter ( $N_{I}^{2}$ in the approximation given in Equation (11)), and therefore, a much steeper early increase occurs in *χ*(*t*) under weak disorder.

In summary, as measured by *χ*(*t*), indeed, disorder slows down the ability of excitons to migrate at very early times, and increasing the Rabi splitting leads to faster initial migration probability for dipoles in photonic wires. Similar considerations can be made on the asymptotic (*t* → ∞) behavior of *χ*(*t*): disorder averaging and the lack of correlation between the excitation energy at distinct sites suppresses cross-terms in the strong disorder limit relative to weak and, therefore, a reduced *σ*_*M*_ favors a larger steady-state value of *χ*(*t*). Additionally, Figure 3a suggests a steady-state time for *χ*(*t*) that is almost independent of disorder. This contrasts with the RMSD behavior shown in Figure 3b, implying that the approximately disorder-independent *χ*(*t*) steady-state times correspond to a feature specific to the nonstandard observable *χ*(*t*). For the rest of this manuscript, we characterize the transport velocity using the RMSD; therefore, we leave a detailed analysis of *χ*(*t*) for future studies.

The RMSD measure reported in Figure 3b also indicates that the excitonic propagation is fastest in the fs time scale. Both *χ*(*t* → ∞) and RMSD(*t* → ∞) drop when the *σ*_*M*_/Ω_*R*_ is increased from 20 % to 40 %. However, comparing the 40 % and 100 % relative disorder strength cases in Figure 3b, we note the emergence of a disorder-enhanced transport regime (DET) as reported in previous studies of dipole chains under strong light–matter interactions [31], [33], [34], [40]. This DET regime clearly leaves no signature in *χ*(*t*) (Figure 3a) but may be understood based on earlier studies of exciton transport in a polaritonic wire. In this regime, weakly coupled states are expected to be exponentially localized but can have slowly decreasing extended tails [31], [34]. These tails carry a small probability away from the initial excitation spot and contribute to the greater RMSD values reported with increasing *σ*_*M*_/Ω_*R*_. Conversely, the extended tails associated with DET leave no signature in *χ*(*t*), as this quantity only tracks the probability in the bulk of the wave packet. These points are corroborated by Figures S3–5 of the SI, where we show the decay profile of wave packets and the emergence of the extended tails responsible for DET.

We reinforce that the asymptotic behavior seen in both Figure 3a and b can be attributed to Anderson localization and not to finite-size artifacts. To demonstrate that, we show in Figure S1 the shape of the wave packets at long propagation times, whereas Figure S2 reveals that, under sufficiently strong disorder, the exciton probability at *x*_0_ ± *L*_*x*_/2 (i.e., where periodic boundary conditions are enforced) remains negligible throughout the simulation.

In Figure 3b, dotted lines represent linear fits obtained from the first 500 fs of simulation. This initial linear behavior (minimum coefficient of determination *R* ^2^ = 0.98) characterizes the excitonic ballistic spread. In the next sections, we will use this value as a measure of the initial exciton spread velocity (*v*_0_). Our choice of a 500-fs time interval aimed to average out complicated features at very early times and to avoid localization effects observed around 1 ps. We checked that all reported qualitative trends are unaffected by a choice of initial time interval that satisfies the described conditions (see Figures S10–12 for a comparison of *v*_0_ obtained using different initial time intervals).

The existence of a short-lived ballistic regime even under strong disorder (*σ*_*M*_/Ω_*R*_ ≥ 1) is a generic feature of sufficiently narrow wave packets. It follows from the fact that the *n*th exciton probability at early times *t* → 0 is given by *P*_*n*_(*t*) ≈ *P*_*n*_(0) + *a*_*n*_ *t* ^2^, where *a*_*n*_ is a site-dependent constant [41]. Therefore, RMSD^2^(*t*) at sufficiently small *t* is equal to RMSD^2^(0) + $\sum_{n}{\left(x_{n}-x_{0}\right)}^{2}a_{n}t^{2}$ . Hence, there is a short time frame where RMSD(*t*) = $\sqrt{{\text{RMSD}}^{2}\left(0\right)+ct^{2}}$ , where $c=\sum_{n}{\left(x_{n}-x_{0}\right)}^{2}a_{n}$ . If RMSD(0) is small enough, then there exists a short time interval RMSD(*t*) is proportional to *t* regardless of the amount of disorder.

To conclude this subsection, we note the polariton-mediated ultrafast exciton transport described here shows intriguing differences relative to bare exciton transport analyzed in a recent study by Cui et al. [35]. Their work showed that transient ultrafast energy transfer mediated by direct short-range interactions benefits from the existence of static disorder, leading to faster transport (relative to a perfectly ordered system) in the femtosecond timescale. Cui et al. ascribe their observation of transient disorder enhancement of transport to the suppression of destructive interference induced by the heterogeneity of the matter excitation energies [35]. Here, we find the opposite feature: disorder always reduces the initial transport velocity. Even in the DET regime, Figure 3b shows a narrow window at early times where transport is subdiffusive [33]. This contrast points toward a fundamental difference in how static disorder affects direct and polariton-mediated coherent exciton transport.

### 3.2 Ballistic exciton transport

**Figure 4:**
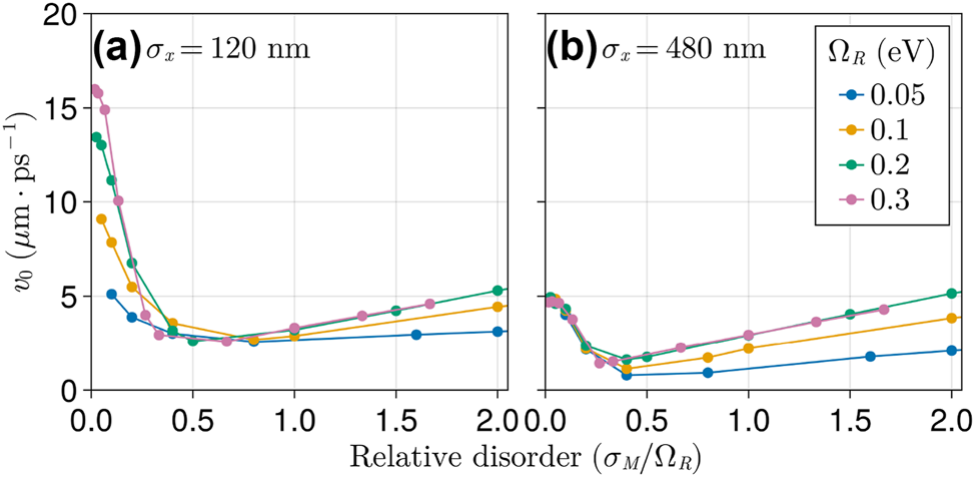
Initial ballistic velocity (*v*_0_) for various wave packets with a (a) narrow and (b) broad initial spread values (*σ*_*x*_, see Equation (6)). *v*_0_ was computed as the slope of a linear fit of RMSD values in the initial 500 fs of simulation (see Figure 3).

In Figure 4, the initial exciton spread velocity (*v*_0_) is shown as a function of relative disorder *σ*_*M*_/Ω_*R*_. We examine *v*_0_ obtained for systems with variable collective light–matter interaction strength Ω_*R*_ (with fixed relative disorder *σ*_*M*_/Ω_*R*_) and two selected initial wave packet sizes (*σ*_*x*_ in Equation (6)). In both cases, we observe an initial steep decay of *v*_0_ with increasing *σ*_*M*_/Ω_*R*_, which is followed by a plateau until the DET regime is reached at *σ*_*M*_/Ω_*R*_ ≈ 1. However, a salient difference in the variation of *v*_0_ with Ω_*R*_ at low disorder is observed between the narrow (*σ*_*x*_ = 120 nm) and the broader (*σ*_*x*_ = 480 nm) wave packets in Figure 4a and b, respectively. This difference vanishes quickly when disorder is increased, demonstrating the initial state preparation is less important to the dynamics under strong disorder. Nevertheless, the distinct Ω_*R*_ dependence of *v*_0_ is observable in a sizable range of disorder strengths (0 ∼ 30 %), thereby warranting a mechanistic explanation. We pursue that by analyzing below the (excitonic) spread velocity of the wave packet in the absence of disorder.

The detailed mathematical treatment of the spread velocity, which we summarize below, is provided in Section 1 of the SI. We first emphasize that the treatment of the ballistic transport regime is unconventional even in the zero-disorder case because (a) our initial wave packets range from strongly localized (*σ*_*x*_ = 120 nm) to moderately delocalized (*σ*_*x*_ = 480 nm) in real space, (b) the wave packet has LP and UP components, and (c) the polariton dispersion is not quadratic. These features imply the basic treatment of Gaussian wave packet transport in a quadratic medium, generally valid for sufficiently narrow wave packets in *q*-space, is inapplicable [42]. With these considerations, we show (see SI) that the dominant contribution to the exciton transport velocity *v*_0_ is given by

(13) $$v_{0}^{2}\approx \underset{q}{\sum }P\left(q\right)\left[{\left(v_{\text{LP}q}^{\text{eff}}\right)}^{2}+{\left(v_{\text{UP}q}^{\text{eff}}\right)}^{2}\right],$$

where *P*(*q*) is the *t* = 0 exciton probability distribution in *q*-space. From Equation (7), the width *σ*_*q*_ of *P*(*q*) is inversely proportional to the real-space width of the initial wave packet *σ*_*x*_. The effective group velocity $v_{\alpha q}^{\text{eff}}$ (where *α* is UP or LP) is defined as

(14) $$v_{\alpha q}^{\text{eff}}=\Pi_{\alpha q}v_{g}^{\alpha q}=\Pi_{\alpha q}\frac{\partial \omega_{\alpha q}}{\partial q},$$

where Π_*αq*_ is the total exciton content of the polariton mode *α* with wave number *q*, and $\frac{\partial \omega_{\alpha q}}{\partial q}$ is the (conventional) group velocity $v_{g}^{\alpha q}$ of the same mode. The polaritonic group velocity weighted by the corresponding exciton content Π_*αq*_ yields the effective exciton group velocity $v_{\alpha q}^{\text{eff}}$ of mode $\left|\alpha q\right\rangle$ . The total matter content Π_*αq*_ plays a key role because even though high energy regions of the UP branch yield the largest $v_{g}^{\alpha q}$ , their small Π_*αq*_ results in negligible $v_{\alpha q}^{\text{eff}}$ values. From Equation (13) one can see that *v*_0_ is controlled by the effective group velocity $v_{\alpha q}^{\text{eff}}$ weighted by the exciton *P*(*q*) distribution. Hence, as we demonstrate below, the different ways *P*(*q*) and $v_{\alpha q}^{\text{eff}}$ overlap explain the varying mobility of differently prepared excitons in weakly and moderately disordered systems.

**Figure 5:**
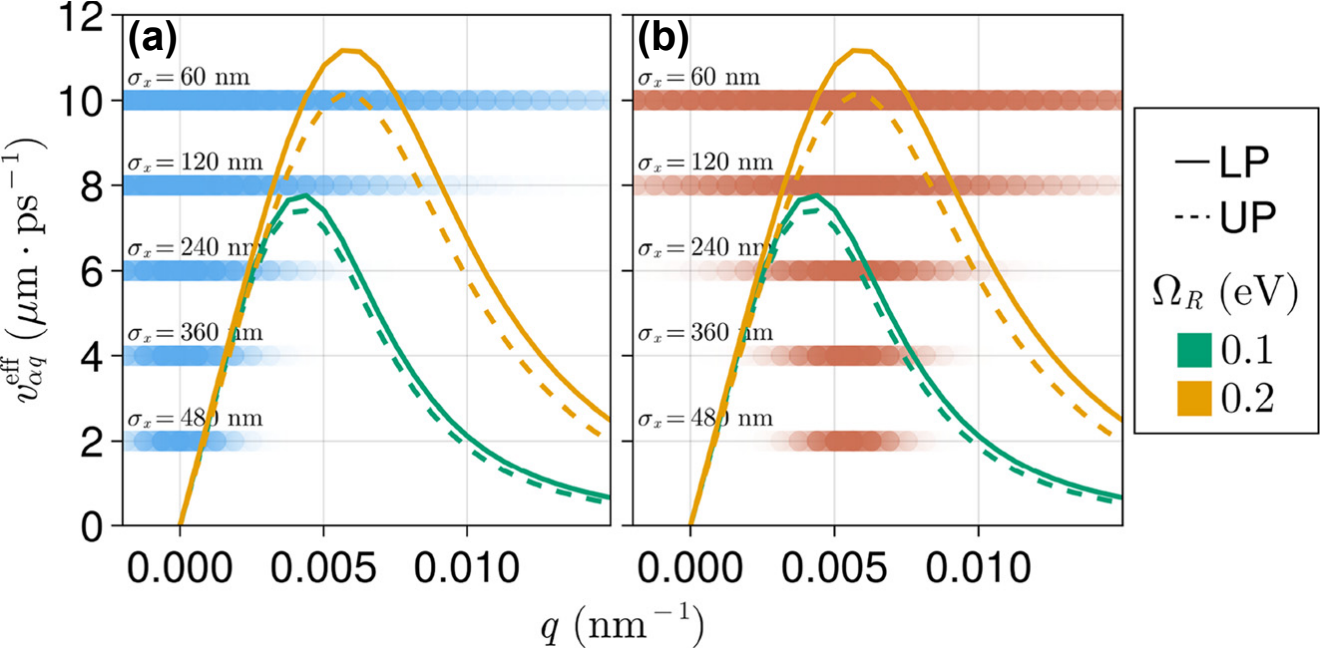
Effective group velocity (Equation (14)) for different values of Ω_*R*_. Horizontal gradient bars represent the *P*(*q*) distribution for different exciton wave packets (see Equation (6)) with (a) ${\bar{q}}_{0}=0.0$ and (b) ${\bar{q}}_{0}=0.0055$ nm^−1^. The overlap between the gradient bars (*P*(*q*)) and the $v_{\alpha q}^{\text{eff}}$ curves yields the exciton ballistic velocity *v*_0_ (Equation (13)). See text for more details.

Effective group velocities $v_{\alpha q}^{\text{eff}}$ for systems with Ω_*R*_ = 0.1 and Ω_*R*_ = 0.2 eV are presented with an overlay of *P*(*q*) for several initial exciton wave packets in Figure 5. The first relevant observation is the substantial increase of $v_{\alpha q}^{\text{eff}}$ with Ω_*R*_ for both *α* = LP and UP with *q* > 0.003 nm^−1^. The Rabi splitting does not affect the effective group velocity for polariton modes with *q* < 0.003 nm^−1^. This feature explains the Rabi splitting dependence of *v*_0_ observed in Figure 4b in weakly and moderately disordered systems. Specifically, a broad wave packet in real space (e.g., *σ*_*x*_ = 480 nm) is narrow in *q*-space and is only non-vanishing at a small interval of *q* near zero (the wave packet center in *q* space) where $v_{\alpha q}^{\text{eff}}$ is nearly identical for all values of Ω_*R*_ examined here. Therefore, as seen in Figure 4b, sufficiently broad wave packets show no significant dependence on Ω_*R*_ when *σ*_*M*_/Ω_*R*_ is not too large (up to 40 % in Figure 4).

**Figure 6:**
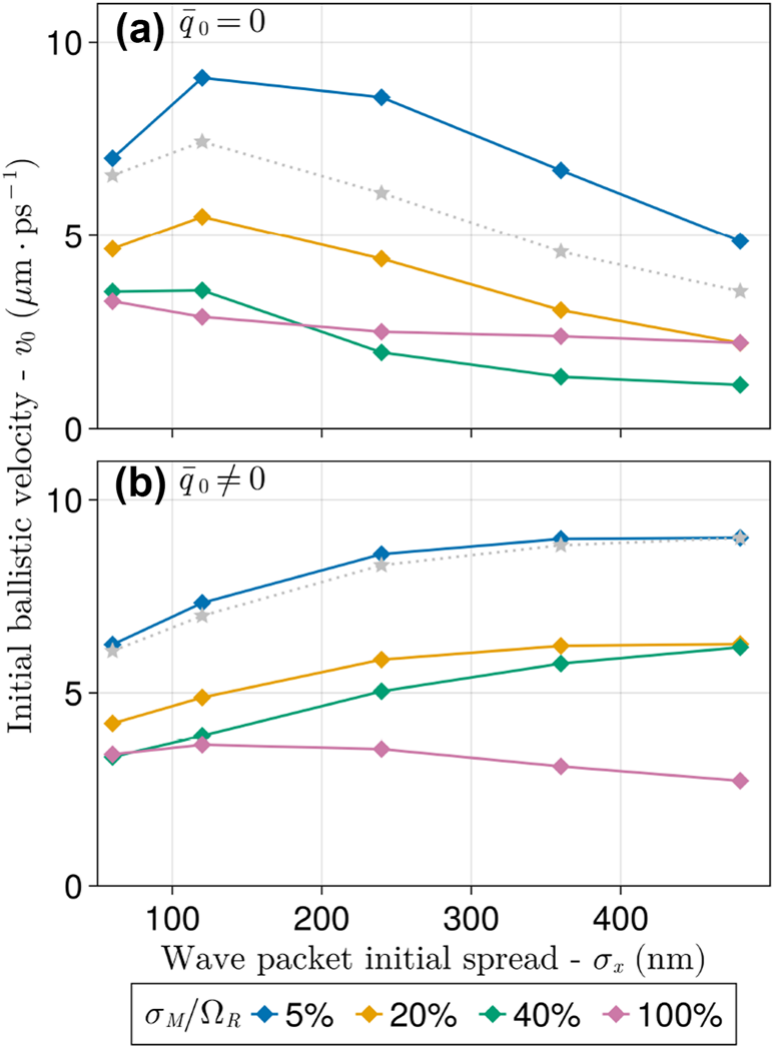
Initial ballistic velocity (*v*_0_) dependency on the wave packet initial spread (*σ*_*x*_) with: (a) ${\bar{q}}_{0}=0$ and (b) ${\bar{q}}_{0}=0.0056$ nm^−1^. Gray dotted curves are values obtained from Equation (13).

In Figure 6, we present quantitative evidence that Equations (13) and (14) appropriately describe the early-time exciton wave packet propagation rate under small and moderate disorder conditions. In particular, Figure 6 shows *v*_0_ versus *σ*_*x*_ at various relative disorder strengths (*σ*_*M*_/Ω_*R*_). As shown in Figure 6a, in almost every case considered, when the initial state has zero average momentum $\left({\bar{q}}_{0}=0\right)$ , an increase in *σ*_*x*_ results in a slower propagation. This generic behavior at weak and moderate disorder can be readily understood from Figure 5a and b. As *σ*_*x*_ broadens, the width of the *P*(*q*) distribution (centered at ${\bar{q}}_{0}=0$ ) is reduced, and *v*_0_ decreases due to the increasing dominance of contributions with small effective group velocities in Equation (13).

The gray dotted curves in Figure 6 follow from Equation (13). This equation not only captures the overall qualitative trend at small and moderate disorder but also reproduces the local maximum at *σ*_*x*_ = 120 nm in Figure 6a. This peak can be explained again based on Figure 5: by increasing the wave packet width in *q*-space, polariton components with larger $v_{\alpha q}^{\text{eff}}$ become relevant and *v*_0_ (Equation (13)) increases, but if *P*(*q*) becomes too broad (e.g., *σ*_*x*_ = 60 nm), polaritons with smaller $v_{\alpha q}^{\text{eff}}$ (with *q* greater than the maxima in $v_{\alpha q}^{\text{eff}}$ ) start to contribute significantly to *v*_0_ (at the expense of high exciton group velocity components) leading to an overall reduction in the magnitude of *v*_0_.

Figure 6b shows analogous results for an exciton prepared with ${\bar{q}}_{0}=0.005654$ nm^−1^. Indeed, in this case, broader wave packets display higher mobility than narrow ones as measured by *v*_0_. This is expected based on Equations (13) and (14) as here a smaller uncertainty in *q* (increased *σ*_*x*_) localizes *P*(*q*) around $q\approx {\bar{q}}_{0}\neq 0$ where the corresponding $v_{\alpha q}^{\text{eff}}$ values are appreciable (see Figure 5b). Overall, these results give a simple prescription to optimize polariton-mediated coherent exciton transport by preparing a sufficiently broad initial state (large *σ*_*x*_ and small *σ*_*q*_) with average momentum ( ${\bar{q}}_{0}$ ) centered at the maximum of $v_{\alpha q}^{\text{eff}}$ .

Note that polariton states no longer have well-defined *q* values in the presence of nonvanishing disorder, and strictly speaking, the arguments above based on uncertainty relations break down. However, in Figure 6, this breakdown is only observed when *σ*_*M*_/Ω_*R*_ = 1.0, where *v*_0_ is nearly independent of *σ*_*x*_. Our analysis in terms of uncertainty relations and Equations (13) and (14) is seen to hold qualitatively for *σ*_*M*_/Ω_*R*_ < 40 %, indicating that the model presented here can be used for examination of coherent exciton transport at early times even in systems with moderate disorder.

To conclude, we investigate the effect of light–matter detuning *δ* = *ℏω*_0_ − *E*_*M*_ on polariton-assisted exciton propagation. This study is motivated by detuning being a simple controllable microcavity parameter [1] and by previous work, which reported greater steady-state exciton migration probability under negative detuning (red-shifted cavities, where *E*_*M*_ > *ℏω*_0_) [43]. We investigate the early dynamics in detuned microcavities by computing *v*_0_ for a variable *E*_*M*_ and fixed cavity lowest-energy mode *ℏω*_0_ = 2.0 eV. To gain insight into the long-time properties of the wave packet, we also show the maximum RMSD value observed over 5 ps.

**Figure 7:**
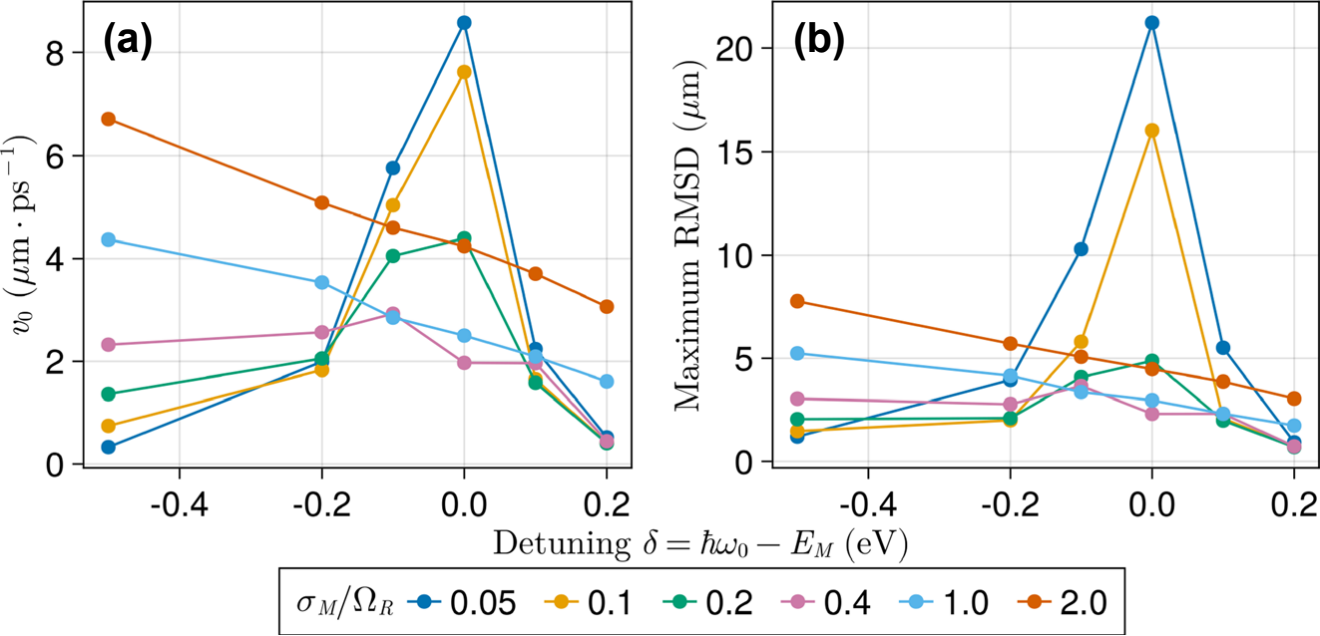
Disorder-dependent detuning effects on coherent exciton transport measured by (a) the initial spread velocity (*v*_0_) and (b) the maximum RMSD over 5 ps. In all cases, Ω_*R*_ = 0.1 eV and *σ*_*x*_ = 240 nm.

**Figure 8:**
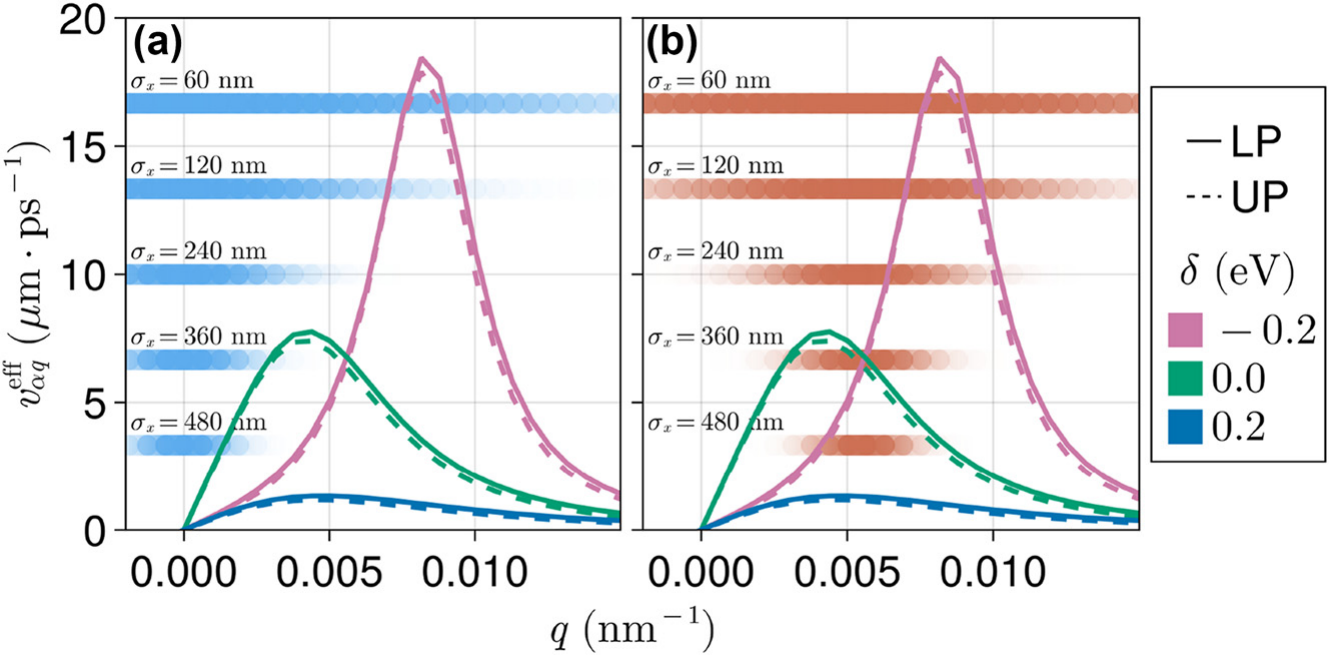
Effective group velocity (Equation (14)) for variable detuning *δ* = *ℏω*_0_ − *E*_*M*_. Horizontal gradient bars represent the *P*(*q*) distribution for distinct exciton wave packets (see Equation (6)) with (a) ${\bar{q}}_{0}=0.0$ and (b) ${\bar{q}}_{0}=0.0055$ nm^−1^. The overlap between the gradient bars (*P*(*q*)) and the $v_{\alpha q}^{\text{eff}}$ curves yields the exciton spread velocity *v*_0_ (Equation (13)). See text for more details.

In Figure 7, we find detuning effects on *v*_0_ and the maximum RMSD are very similar. Under weak disorder (*σ*_*M*_/Ω_*R*_ < 0.4), both *v*_0_(*δ*) and RMSD(*δ*) are peaked at *δ* = 0, i.e., the coherent exciton motion is faster when the cavity is in resonance with the dipolar excitation. This can be rationalized with Figure 8a, which shows a strong dependence of $v_{\alpha q}^{\text{eff}}$ on detuning. Blue-shifted microcavities lead to the slowest exciton motion as evidenced by the consistently smaller $v_{\alpha q}^{\text{eff}}$ obtained for *δ* = 0.2 eV in Figure 8. On the other hand, red-shifted microcavities have small $v_{\alpha q}^{\text{eff}}$ at *q* close to zero but higher values (compared to the resonant cavity) at sufficiently large *q*. Since the initial wave packets of Figure 7 have ${\bar{q}}_{0}=0$ , the dominant polariton contributions to the evolution are those for which $v_{\alpha q}^{\text{eff}}$ is larger at zero-detuning in comparison to the red-shifted case. Nevertheless, comparison between $v_{\alpha q}^{\text{eff}}$ at zero and negative detuning in Figure 8b suggests that polariton-mediated exciton wave packet transport can be much faster in red-shifted cavities when the initial-state is prepared with ${\bar{q}}_{0}$ close to the maximum of $v_{\alpha q}^{\text{eff}}$ . Indeed, we show numerical results in our supporting information (Figure S13) that confirm this prediction.

In the presence of stronger disorder (*σ*_*M*_ > 0.4Ω_*R*_), Figure 7 shows the condition *δ* = 0 no longer provides a maximum RMSD and *v*_0_, and the optimal detuning value is shifted toward negative values. Therefore, under sufficient disorder, red-shifting the microcavity enhances the exciton ballistic transport and the transport distance regardless of the initial wave packet preparation. This feature may be understood by noting that in the presence of a significant amount of static disorder, many dipoles will have excitation energies below the lowest-energy microcavity mode. In this case, it becomes advantageous to employ negative detuning, since it supports lower energy photon modes that can interact resonantly with the dipoles with lower excitation energy. Conversely, raising *ℏω*_0_ − *E*_*M*_ to positive values leads to a reduction in the light–matter spectral overlap and, therefore, the maximum RMSD and *v*_0_ consistently decrease as the microcavity is blue-shifted (*δ* > 0). Despite an optimum not being visible in Figure 7, we show in Figure S14 that both *v*_0_ and the maximum RMSD reach a plateau between *δ* = −1 eV and *δ* = −2 eV. However, we highlight that under such extreme values of negative detuning, the energy gap between the dipole excitation (*E*_*M*_) and higher photonic bands (e.g., *n*_*y*_ = 1, *n*_*z*_ = 2 and vice-versa) is about the same as the gap between the dipole excitation and the lowest photon mode (*q* = 0, *n*_*y*_ = *n*_*z*_ = 1). In this case, higher energy photon bands may start contributing to the dynamics just as much as EM modes in the *n*_*y*_ = *n*_*z*_ = 1 band. Therefore, a systematic analysis at large negative detunings must include multiple microcavity bands. We present these results here as they show a well-defined theoretical limit for the employed single-band model.

### 3.3 Losses and dynamical disorder

We finalize our discussion by offering brief considerations of how the exciton transport phenomena examined in this article would be affected by photon leakage and dynamical disorder (e.g., represented by time-dependent stochastic fluctuations of excitonic transition energies, dipole orientations, or intersite distances).

Photon leakage through imperfect mirrors is an unavoidable characteristic of physics in optical microcavities [1]. However, the impact of photon losses on the polariton-assisted exciton transport discussed here is minimal, especially at moderate and large disorder. Under these conditions, the typical time-dependent photon content is small enough (see Figure 2 and S1) that wave packet decay by photon loss is a slow process. For example, if the empty cavity photon lifetime is 50 fs, the corresponding wave packet lifetime is at least one or two orders of magnitude bigger, depending on its photonic content. Therefore, while cavity leakage could prevent observation of DET and subsequent Anderson localization in moderate and strongly disordered systems, the ultrafast ballistic regime would be almost unaffected.

In the weakly disordered scenario where photon content fluctuations can reach a significant value (Figure 2 and S1), the fast decay of the wave packet would prevent efficient energy transport. Nevertheless, even in this case, incorporation of cavity losses is unlikely to change the main qualitative trends observed for *v*_0_ as this quantity is computed at ultrafast times (e.g., Figures S10–12 show that the behavior of *v*_0_ with varying disorder and Rabi splitting is independent of the linear fit cutoff time).

The interplay between static and dynamical disorder is an interesting topic that we aim to address in detail in future work. Here, we simply point out some expected effects of dynamical disorder on the examined polariton-assisted exciton transport. First, dynamical disorder suppresses Rabi oscillations and may slow down the relatively efficient ultrafast exciton transport we observe at weak disorder. On the other hand, recent work by Cui and Nitzan has shown that dynamical disorder boosts the early-time exciton transport [35]. Further, it is well known that dynamical disorder prevents Anderson localization and leads to diffusive behavior at long times [41]. The latter behavior is generic so we expect it to persist in the moderate and strong (static) disorder regimes of polariton-assisted exciton transport. A recent study [32] suggests that excitonic interaction with a dynamical (finite-temperature) environment shortens the ballistic transport regime time interval and leads to a quick crossover to diffusive behavior. We expect similar features will emerge when dynamical disorder is incorporated into our model, but future work is necessary to determine the interplay between the effects of static and dynamical disorder in the investigated polariton-assisted exciton transport.

## 4 Conclusions

We examined coherent polariton-mediated exciton transport on a lossless disordered polaritonic wire. Our analysis shows that the initial exciton wave packet (i.e., its spread and average momentum) strongly influences its ballistic propagation regime and may be optimized to maximize its early mobility. A striking contrast between polariton-mediated and purely excitonic transport was also noted here. Previous work showed that short-time direct exciton energy transport (via dipole–dipole interactions) is enhanced by disorder [35]. Here, we find, contrarily, that disorder systematically suppresses the initial wave packet spread. This implies a fundamental distinction in how disorder impacts coherent exciton energy transport inside and outside an optical microcavity.

We also analyzed the interplay of detuning and static disorder as factors impacting the ballistic transport regime. We found that while blue-shifted cavities always presented a slower exciton wave packet transport, red-shifted microcavities showed richer behavior, i.e., both suppression and enhancement of transport can be attained depending on the level of disorder and the initial state preparation.

To rationalize these results, we introduced the effective exciton group velocity $v_{\alpha q}^{\text{eff}}$ , which can be computed from the system dispersion and the excitonic content of each polariton eigenmode. The early ballistic transport can be estimated by combining $v_{\alpha q}^{\text{eff}}$ with the initial exciton probability distribution in *q* space. This analysis leads to a design principle for optimizing the initial exciton state for enhanced ultrafast coherent transport based on the complex interplay between disorder and tunable light–matter parameters such as detuning, Rabi splitting, and initial wave packet width and momentum. The optimal initial state for exciton transport must have (i) an initial wave vector matching the maximum value of the effective exciton group velocity and (ii) a sufficiently narrow spread in *q*-space such that it only spans eigenmodes with large effective exciton group velocity.

Our theoretical analysis of exciton wave packet propagation in terms of the newly introduced effective exciton group velocity led to qualitative agreement with simulations even under moderate disorder (*σ*_*M*_/Ω_*R*_ = 0.4). Such robustness and generalizability suggest our results will be useful for future theoretical and experimental studies of transport in optical cavities.

## Supplementary Material

Supplementary Material Details
