# Supplementary material for: Coherent transient exciton transport in disordered polaritonic wires
Source: Nanophotonics. 2024 Feb 21;13(14):2553–64. doi: 10.1515/nanoph-2023-0797 (PMC11636474; doi:10.1515/nanoph-2023-0797)
Supplement: Supplementary file 1 — Supplementary Material Details [file j_nanoph-2023-0797_suppl_001.pdf]

Supporting Information

Coherent transient exciton transport in disordered polaritonic wires

Gustavo J. R. Aroeira, Kyle T. Kairys, and Raphael F. Ribeiro*

Department of Chemistry and Cherry Emerson Center for Scientific Computation, Emory University, Atlanta, Georgia 30322, United States of America

E-mail: raphael.ribeiro@emory.edu

Table S1: List of symbols and notation used in this work

Symbol	Description	Value
N_M	Number of dipoles in the wire.	5000
N_c	Number of cavity modes used to describe the radiation field inside the cavity.	1001
Ω_R	Rabi splitting: a measure of the collective light-matter interaction strength.	Variable
a	Intersite distance.	10 nm
σ_a	Standard deviation of sites positions.	1 nm
E_M	Dipole excitation energy.	Variable
σ_M	Standard deviation of the distribution of dipole excitation energies.	Variable
L_z	Wire length along z dimension.	0.4 μm
L_y	Wire length along y dimension.	0.2 μm
L_x	Wire length along x dimension.	50 μm
ϵ	Relative static permittivity.	3
n_y	Cavity quantum number associated with the y dimension.	1
n_z	Cavity quantum number associated with the z dimension.	1
m_x	Cavity quantum number associated with the x dimension.	$\in \mathbb{Z}$
q	x-component of the wavevector $\mathbf{k}$	$2\pi m_x/L_x$.
σ_x	Initial wave packet width.	Variable
$\bar{q}_0$	Average initial exciton momentum along x	Variable

Contents

List of Figures **S3**

1 RMSD for a traveling exciton-polariton **S6**

1.1 Preliminary expressions S6

1.1.1 Wave packet representations S6

1.1.2 Polariton Eigenstates S7

1.1.3 Continuum limit S8

1.1.4 Time-evolved wave packet S10

1.2 Exciton mean squared displacement S10

2 Exciton migration probability **S14**

3 Wave packets at long propagation times **S17**

4 Wave packets under DET **S19**

5 Propagation Profiles **S21**

6 Linear Fit Range **S26**

7 v_0 for detuned cavities **S28**

8 Strong Coupling Signatures **S31**

List of Figures

S1 Average exciton wave packet profiles at different time delays and relative disorder strength (σ_M/Ω_R) of 5%, 20%, and 100% for (a), (b) and (c), respectively. In all cases, $\Omega_R = 0.1$ eV and the wave packet initial width and mean quasimomentum are $\sigma_x = 120$ nm and $\bar{q}_0 = 0$. Probabilities are grouped in bins containing 50 dipoles spanning 0.5 μm. P_M, RMSD, and χ are defined in Equations (74),(38), and (76), respectively. Each profile is obtained from the average of 100 disorder realizations. S17

S2 Probability of finding the exciton near the boundary of the wire. This value is computed by summing the excitation probability of **(a)** 100 and **(b)** 500 sites closest to the region of the wire where periodic boundary conditions are enforced ($x = 0$ and $x = 50$ μm). In all cases, $\Omega_R = 0.1$ eV and the wave packet initial width and mean quasimomentum are $\sigma_x = 120$ nm and $\bar{q}_0 = 0$. Each data point is obtained from the average of 100 disorder realizations. . . . S18

S3 Wave packet properties at $t = 500$ fs: **(a)** Decay as a function of distance at different relative disorder strengths. **(b)** RMSD and migration probabilities at a fixed time step at different disorder strengths. In all cases, $\Omega_R = 0.1$ eV and $\sigma_x = 120$ nm. Other parameters are fixed as in the main text. S19

S4 Wave packet properties at $t = 1000$ fs: **(a)** Decay as a function of distance at different relative disorder strengths. **(b)** RMSD and migration probabilities at a fixed time step at different disorder strengths. In all cases, $\Omega_R = 0.1$ eV and $\sigma_x = 120$ nm. Other parameters are fixed as in the main text. S19

S5 Wave packet properties at $t = 2000$ fs: **(a)** Decay as a function of distance at different relative disorder strengths. **(b)** RMSD and migration probabilities at a fixed time step at different disorder strengths. In all cases, $\Omega_R = 0.1$ eV and $\sigma_x = 120$ nm. Other parameters are fixed as in the main text. S20

S6 Propagation of exciton wave packets at different disorder strengths measured by **(a)** migration probability (Eq. 76) and **(b)** RMSD (Eq. 74). In all cases, $\sigma_x = 120$ nm and $\Omega_R = 0.05$ eV. Results are averages of 100 disordered realizations. The dotted lines in **(b)** are linear fits on the early propagation ($\leq$ 500 fs) from which slopes are used to measure the initial ballistic velocity (v_0). Panels **(c)** and **(d)** are zoomed in versions of **(a)** and **(b)**, respectively.S22

S7 Propagation of exciton wave packets at different disorder strengths measured by **(a)** migration probability (Eq. 76) and **(b)** RMSD (Eq. 74). In all cases, $\sigma_x = 120$ nm and $\Omega_R = 0.1$ eV. Results are averages of 100 disordered realizations. The dotted lines in **(b)** are linear fits on the early propagation ($\leq$ 500 fs) from which slopes are used to measure the initial ballistic velocity (v_0). Panels **(c)** and **(d)** are zoomed in versions of **(a)** and **(b)**, respectively. S23

S8 Propagation of exciton wave packets at different disorder strengths measured by **(a)** migration probability (Eq. 76) and **(b)** RMSD (Eq. 74). In all cases, $\sigma_x = 120$ nm and $\Omega_R = 0.2$ eV. Results are averages of 100 disordered realizations. The dotted lines in **(b)** are linear fits on the early propagation ($\leq$ 500 fs) from which slopes are used to measure the initial ballistic velocity (v_0). Panels **(c)** and **(d)** are zoomed in versions of **(a)** and **(b)**, respectively. S24

S9 Propagation of exciton wave packets at different disorder strengths measured by **(a)** migration probability (Eq. 76) and **(b)** RMSD (Eq. 74). In all cases, $\sigma_x = 120$ nm and $\Omega_R = 0.3$ eV. Results are averages of 100 disordered realizations. The dotted lines in **(b)** are linear fits on the early propagation ($\leq$ 500 fs) from which slopes are used to measure the initial ballistic velocity (v_0). Panels **(c)** and **(d)** are zoomed in versions of **(a)** and **(b)**, respectively. S25

S10 Initial spread velocity (v_0) for various systems at different relative disorder strength and Rabi splitting values for wave packets with a **(a)** narrow and **b** broad initial spread values (σ_x). Each point is the average of 100 disordered realizations. v_0 was computed as the slope of a linear fit of RMSD values in the initial 100 fs of simulation (see Figure 3) in the main text. S26

S11 Initial spread velocity (v_0) for various systems at different relative disorder strength and Rabi splitting values for wave packets with a **(a)** narrow and **b** broad initial spread values (σ_x). Each point is the average of 100 disordered realizations. v_0 was computed as the slope of a linear fit of RMSD values in the initial 300 fs of simulation (see Figure 3) in the main text. S27

S12 Initial spread velocity (v_0) for various systems at different relative disorder strength and Rabi splitting values for wave packets with a **(a)** narrow and **b** broad initial spread values (σ_x). Each point is the average of 100 disordered realizations. v_0 was computed as the slope of a linear fit of RMSD values in the initial 700 fs of simulation (see Figure 3) in the main text. S27

S13 Ballistic velocity (v_0) as a function of the cavity detuning (δ) for several disorder strengths (σ_M/Ω_R). Left column panels (a, c, e, g) show results for excitons with zero quasi-momentum ($\bar{q}_0 = 0$) whereas in the right column panels excitons have $\bar{q}_0 = 0.008$ nm^{-1}. In each panel, a different initial spread is used (σ_x). S29

S14 Disorder-dependent detuning effects on coherent exciton transport measured by **(a)** the ballistic velocity (v_0), and **(b)** the maximum RMSD over 5 ps. In all cases $\sigma_x = 240$ nm and $\Omega_R = 0.1$ eV. Each data point corresponds to an average over 100 disorder realizations. S30

S15 Average energy vs photon q with maximum probability for bright modes (total photon content $> 10\%$) of systems under increasing disorder. Each point represent the mean energy obtained from the eigenstates with more than 10% photonic content in 25 realizations of the light-matter Hamiltonian with the specified σ_M/Ω_R and Ω_R fixed at 0.1 eV. For the sake of simplicity, we show results for systems containing $N_M = 1000$ molecules and 401 photonic modes. The graphs show the gradual closing of the polariton gap (here represented by $E_{\mathrm{UP},q} - E_{\mathrm{LP},q}$ with $q = 0$) as σ_M/Ω_R increases. Finer features (e.g., the number of points in the LP and UP belonging to specific intervals of q) are unlikely to be converged due to the small system size and the number of realizations we employed. However, this lack of convergence is irrelevant to our analysis of the polariton gap. S32

S16 Demonstration of the effect of disorder on Rabi oscillations. The excitonic probability is the summed probability that any site is in its excited state. In all cases the Rabi splitting is fixed at 0.1 eV and each point is the average of 100 realizations. S33

1 RMSD for a traveling exciton-polariton

1.1 Preliminary expressions

1.1.1 Wave packet representations

Before we proceed to our main problem, let us establish some intermediate results that will be useful later. First, consider the wave packet at $t = 0$ represented in the position basis of N_M dipoles

$$|\psi_0\rangle = \sum_{n=1}^{N_M} c_n |n\rangle \ . \tag{1}$$

Therefore, the probability of finding the n-th dipole excited is $|c_n|^2$. Note that in the main text, we use the notation $|n; 0\rangle$ to represent the state where the n-th dipole is excited and no photon modes are populated. For simplicity, we use $|n; 0\rangle \rightarrow |n\rangle$ here as the photonic degrees of freedom will not appear explicitly in the following expressions. We can transform $|\psi_0\rangle$ into its k-space representation using the resolution of the identity (in the exciton Hilbert space) $\sum_k |k\rangle\langle k| = \mathbb{1}_E$. That is,

$$|\psi_0\rangle = \sum_k \sum_{n=1}^{N_M} c_n \langle k|n\rangle |k\rangle \ , \tag{2}$$

$$= \sum_k \left(\frac{1}{\sqrt{N_M}} \sum_{n=1}^{N_M} c_n e^{-ikx_n} \right) |k\rangle \ , \tag{3}$$

where we have used $\langle k|n\rangle = \frac{e^{-ikx_n}}{\sqrt{N_M}}$. Defining

$$c_k = \frac{1}{\sqrt{N_M}} \sum_{n=1}^{N_M} c_n e^{-ikx_n} \tag{4}$$

we have the wave function in k-representation

$$|\psi_0\rangle = \sum_k c_k |k\rangle \ . \tag{5}$$

From the expression above, we see that the initial exciton probability distribution in wave number space is $P_k = |c_k|^2$.

1.1.2 Polariton Eigenstates

In the case where the dipole ensemble is translationally invariant, we can assign each eigenmode to the upper polariton (UP) branch if its energy is above the dipolar transition energy or lower polariton (LP) otherwise. The general Hamiltonian for the system can be block diagonalized using the transformation in Eq. 4. The resulting Hamiltonian is a direct sum of 2×2 Hamiltonians where the k wave number is preserved, that is, there is no mixing of different values of k. Using q for the wave number of the polariton states we can write

$$\hat{H} |\alpha q\rangle = \hbar\omega_{\alpha q} |\alpha q\rangle \ , \tag{6}$$

where α is LP or UP. Moreover, the amplitude of the n-th dipole on the eigenstate $|\alpha q\rangle$ is

$$\langle n|\alpha q\rangle = -i\sqrt{\frac{\Pi_{\alpha q}}{N_M}} e^{iqx_n} \ , \tag{7}$$

where $\Pi_{\alpha q} = \sum_{n=1}^{N_M} \langle \alpha q|n\rangle \langle n|\alpha q\rangle$ is the total exciton content of the eigenstate $|\alpha q\rangle$. With this result, the overlap of each eigenstate with the initial wave packet is given by

$$\langle \psi_0|\alpha q\rangle = \sum_{n=1}^{N_M} c_n^* \langle n|\alpha q\rangle \tag{8}$$

$$= -i\sqrt{\Pi_{\alpha q}} \left(\frac{1}{\sqrt{N_M}} \sum_{n=1}^{N_M} c_n^* e^{iqx_n} \right) \ . \tag{9}$$

The term in brackets above is the complex conjugate of Eq. (4). Thus,

$$\langle \psi_0|\alpha q\rangle = -i\sqrt{\Pi_{\alpha q}} c_q^* \ . \tag{10}$$

The resolution of the identity in the eigenmode basis is given by

$$\mathbb{1} = \sum_\alpha \sum_q |\alpha q\rangle \langle \alpha q| \ . \tag{11}$$

1.1.3 Continuum limit

In what follows, we will take the thermodynamic limit where L and N_M go to infinity at the same rate, so L/N_M is fixed. This will lead to closed-form expressions for excitonic observables in the absence of disorder. We will assume throughout that all relevant functions (of the dipole position $x_n = na$ and wavenumber q) are sufficiently slowly varying at the scale of the spatial lattice constant $x_{n+1} - x_n = \Delta x = a$ and reciprocal lattice spacing $\Delta q = 2\pi/L$ so the continuum limit is well defined. We also assume the wave packet vanishes sufficiently fast outside a finite closed subset of position or wave number space. Under these conditions, any sum over discrete functions of the dipole position x_n can be replaced by an integral over all space following

$$\sum_{n=1}^{N_M} f_n \to \frac{1}{a} \int f(x) \mathrm{d}x, \tag{12}$$

where $f(x)$ is the continuum representation of f_n satisfying $f(x_n) = f_n$. Likewise, the continuum limit for sums of discrete functions of q is obtained from

$$\sum_q g_q \to \frac{L}{2\pi} \int g(q) \mathrm{d}q, \tag{13}$$

where $g(q)$ is the continuum representation of g_q.

The continuum limit of the resolution of the identity in the eigenmode basis is given by

$$\mathbb{1} = \frac{L}{2\pi} \sum_\alpha \int |\alpha q\rangle \langle \alpha q| \, dq \tag{14}$$

Thus, it is convenient to redefine $|\alpha q\rangle$ following

$$|\alpha q\rangle \to \sqrt{\frac{2\pi}{L}} \, |a q\rangle \,, \tag{15}$$

so as to recover the identity operator in the standard form

$$\mathbb{1} = \sum_\alpha \int |\alpha q\rangle \langle \alpha q| \, dq \tag{16}$$

Similar manipulations can be performed for the states living in the matter Hilbert space, e.g.,

$$\mathbb{1}_E = \sum_{n=1}^{N_M} |n\rangle\langle n| \to \frac{1}{a}\int |x\rangle\langle x|\,\mathrm{d}x, \tag{17}$$

$$\mathbb{1}_E = \sum_q |q\rangle\langle q| \to \frac{L}{2\pi}\int |q\rangle\langle q|\,\mathrm{d}q. \tag{18}$$

These identities suggest redefining the matter states in the continuum limit following

$$|n\rangle \to \sqrt{a}\,|x\rangle\,, \tag{19}$$

$$|q\rangle \to \sqrt{\frac{2\pi}{L}}\,|q\rangle\,. \tag{20}$$

Using these relations, it follows in the continuum limit

$$\langle k|x\rangle = \sqrt{\frac{L}{2\pi a}}\,\langle k|n\rangle = \frac{e^{-ikx}}{\sqrt{2\pi}}, \tag{21}$$

From these results, we obtain the wave packet representation in position and wave number space analogous to those of Sec. 1.1.1.

$$|\psi_0\rangle = \int c(x)\,|x\rangle\,\mathrm{d}x, \quad c(x) = \langle x|\psi_0\rangle \tag{22}$$

$$|\psi_0\rangle = \int\int c(x)\,\langle q|x\rangle\ \ |k\rangle\,\mathrm{d}k\mathrm{d}x = \int c(k)\,|k\rangle\,\mathrm{d}k, \tag{23}$$

where the wave vector amplitude in the continuous wave number space is given by

$$c(k) = \frac{1}{\sqrt{2\pi}}\int c(x)e^{-ikx}\mathrm{d}x. \tag{24}$$

By performing the analogous transformations to the polariton eigenmodes we obtain

$$\langle x|\alpha q\rangle = -i\sqrt{\frac{\Pi_{\alpha q}}{2\pi}}e^{iqx}, \tag{25}$$

$$\langle\psi_0|\alpha q\rangle = -i\sqrt{\Pi_{\alpha q}}c^*(q). \tag{26}$$

1.1.4 Time-evolved wave packet

The wave packet at an arbitrary time $|\psi_t\rangle$ can be written in terms of the initial state using the time-evolution operator

$$|\psi_t\rangle = e^{-i\hat{H}t/\hbar} |\psi_0\rangle \ . \tag{27}$$

Using the eigenstates discussed in the previous section, in particular Eq. 16, we can resolve the time evolution as

$$|\psi_t\rangle = \sum_\alpha \int_{-\infty}^{+\infty} e^{-i\hat{H}t/\hbar} |\alpha q\rangle \langle \alpha q|\psi_0\rangle \, dq \ . \tag{28}$$

Using Eq. 6 and 10 we get

$$|\psi_t\rangle = i \sum_\alpha \int_{-\infty}^{+\infty} \sqrt{\Pi_{\alpha q}} c(q) e^{-i\omega_{\alpha q} t} |\alpha q\rangle \, dq \ . \tag{29}$$

1.2 Exciton mean squared displacement

The exciton mean squared displacement Δx_E^2 can be written as

$$\Delta x_E^2(t) = \frac{1}{P_M(t)} \int |\langle x|\psi_t\rangle|^2 (x - x_0)^2 \mathrm{d}x \tag{30}$$

$$P_M(t) = \int |\langle x|\psi_t\rangle|^2 \mathrm{d}x \ , \tag{31}$$

where x_0 is the center of the initial wave packet. We start by computing $P_M(t)$ from the inner product of $|x\rangle$ with $|\psi_t\rangle$ (Eq. 29). The result is

$$\langle x|\psi_t\rangle = \frac{1}{\sqrt{2\pi}} \sum_\alpha \int_{-\infty}^{+\infty} \Pi_{\alpha q} c(q) e^{-i\omega_{\alpha q} t} e^{iqx} dq \ . \tag{32}$$

Hence,

$$P_M = \int \langle \psi_t|x\rangle \langle x|\psi_t\rangle \, \mathrm{d}x \tag{33}$$

$$= \frac{1}{2\pi} \sum_\alpha \sum_\beta \int_{-\infty}^{+\infty} \int_{-\infty}^{+\infty} c^*(q') c(q) \Pi_{\beta q'} \Pi_{\alpha q} e^{-i(\omega_{\alpha q} - \omega_{\beta q'})t} \left(\int e^{i(q-q')x} dx \right) dq dq' \ . \tag{34}$$

Using $2\pi\delta(q-q') = \int \exp[i(q-q')x]dx$, we obtain

$$P_M = \sum_\alpha \sum_\beta \int_{-\infty}^{+\infty} |c(q)|^2 \Pi_{\beta q} \Pi_{\alpha q} e^{-i(\omega_{\alpha q} - \omega_{\beta q})t} dq \ . \tag{35}$$

The double sum over α and β produces four terms. Those are

$$P_M = \int_{-\infty}^{+\infty} |c(q)|^2 \left(\Pi^2_{\mathrm{LP}q} + \Pi^2_{\mathrm{UP}q} + \Pi_{\mathrm{LP}q}\Pi_{\mathrm{UP}q} e^{-i(\omega_{\mathrm{LP}q} - \omega_{\mathrm{UP}q})t} + \Pi_{\mathrm{LP}q}\Pi_{\mathrm{UP}q} e^{-i(\omega_{\mathrm{UP}q} - \omega_{\mathrm{LP}q})t} \right) dq \tag{36}$$

$$= \int_{-\infty}^{+\infty} |c(q)|^2 \left(\Pi^2_{\mathrm{LP}q} + \Pi^2_{\mathrm{UP}q} + 2\Pi_{\mathrm{LP}q}\Pi_{\mathrm{UP}q} \cos\left[(\omega_{\mathrm{UP}q} - \omega_{\mathrm{LP}q})t\right] \right) dq \ . \tag{37}$$

As described in the main text, we measure the exciton spread velocity (v_0) by using a linear fit over the initial 500 fs. This process averages out the oscillating terms observed above. From now on, we ignore the time-dependent fluctuations of $P_M(t)$ and work with the more relevant time-averaged exciton content

$$\bar{P}_M = \int_{-\infty}^{+\infty} |c(q)|^2 \left(\Pi^2_{\mathrm{LP}q} + \Pi^2_{\mathrm{UP}q} \right) dq \ . \tag{38}$$

Next, we analyze the remaining part of Δx_E^2. For the sake of simplicity and without loss of generality we assume $x_0 = 0$. We can use the results from Eq. 32 to write

$$P_M \Delta x_E^2(t) = \int |\langle x|\psi_t\rangle|^2 x^2 dx \tag{39}$$

$$= \frac{1}{2\pi} \sum_\alpha \sum_\beta \int_{-\infty}^{+\infty} \int_{-\infty}^{+\infty} c^*(q')c(q)\Pi_{\beta q'}\Pi_{\alpha q} e^{-i(\omega_{\alpha q} - \omega_{\beta q'})t} \left(\int x^2 e^{i(q-q')x} dx \right) dq dq' \ . \tag{40}$$

Rearranging the integrals and using the substitution $x^2 e^{i(q-q')x} = -\frac{\partial^2}{\partial q^2} e^{i(q-q')x}$, we obtain

$$P_M \Delta x_E^2(t) = -\frac{1}{2\pi} \sum_\alpha \sum_\beta \int_{-\infty}^{+\infty} c(q)\Pi_{\alpha q} e^{-i\omega_{\alpha q}t} \left(\int_{-\infty}^{+\infty} c^*(q')\Pi_{\beta q'} e^{i\omega_{\beta q'}t} \frac{\partial^2}{\partial q^2} \left(\int e^{i(q-q')x} dx \right) dq' \right) dq \tag{41}$$

$$= -\sum_\alpha \sum_\beta \int_{-\infty}^{+\infty} c(q)\Pi_{\alpha q} e^{-i\omega_{\alpha q}t} \left(\int_{-\infty}^{+\infty} c^*(q')\Pi_{\beta q'} e^{i\omega_{\beta q'}t} \frac{\partial^2}{\partial q^2} \delta(q-q') dq' \right) dq \tag{42}$$

Using the smoothness and compact support assumptions specified in our discussion of the continuum limit, it follows that

$$\int_{-\infty}^{+\infty} f(q') \frac{\partial^2}{\partial q^2} \delta(q-q') dq' = \int_{-\infty}^{+\infty} \frac{\partial^2 f(q')}{\partial q^2} \delta(q-q') dq' = \frac{\partial^2 f(q)}{\partial q^2} \ . \tag{43}$$

Therefore, we can reduce Eq. 42 to the form

$$P_M \Delta x_E^2(t) = -\sum_\alpha \sum_\beta \int_{-\infty}^{+\infty} c(q)\Pi_{\alpha q} e^{-i\omega_{\alpha q}t} \frac{\partial^2}{\partial q^2}\left[c^*(q)\Pi_{\beta q} e^{i\omega_{\beta q}t}\right] dq \; . \tag{44}$$

To proceed, we evaluate the second derivative with respect to q

$$\begin{aligned} e^{-i\omega_{\beta q}t} \frac{\partial^2}{\partial q^2}\left[c^*(q)\Pi_{\beta q} e^{i\omega_{\beta q}t}\right] &= e^{-i\omega_{\beta q}t} \frac{\partial}{\partial q}\left[\frac{\partial c^*(q)\Pi_{\beta q}}{\partial q} e^{i\omega_{\beta q}t} + -i\frac{\partial \omega_{\beta q}}{\partial q} t c^*(q)\Pi_{\beta q} e^{i\omega_{\beta q}t}\right] \\ &= \frac{\partial^2}{\partial q^2}[c^*(q)\Pi_{\beta q}] - 2it\frac{\partial \omega_{\beta q}}{\partial q}\frac{\partial}{\partial q}[c^*(q)\Pi_{\beta q}] - tc^*(q)\Pi_{\beta q}\left[i\frac{\partial^2 \omega_{\beta q}}{\partial q^2} + t\left(\frac{\partial \omega_{\beta q}}{\partial q}\right)^2\right]. \end{aligned} \tag{45}$$

To obtain a more compact expression, we define

$$\Delta_{\beta\alpha}(q) = \omega_{\beta q} - \omega_{\alpha q}, \tag{46}$$

$$\gamma_{\alpha q} = c(q)\Pi_{\alpha q}, \tag{47}$$

$$\gamma^*_{\beta q} = c^*(q)\Pi_{\beta q}, \tag{48}$$

$$v_g^{\beta q} = \frac{\partial \omega_{\beta q}}{\partial q}. \tag{49}$$

Using these definitions and plugging Eq. 45 into Eq. 44 we get

$$P_M \Delta x_E^2(t) = -\sum_\alpha \sum_\beta \int_{-\infty}^{+\infty} \left(\frac{\partial^2 \gamma^*_{\beta q}}{\partial q^2} - it\frac{\partial v_g^{\beta q}}{\partial q}\gamma^*_{\beta q} - 2iv_g^{\beta q}t\frac{\partial\, \gamma^*_{\beta q}}{\partial q} - (v_g^{\beta q}t)^2\gamma^*_{\beta q}\right)\gamma_{\alpha q} e^{i\Delta_{\beta\alpha}(q)t} dq \tag{50}$$

Since our goal is to obtain the term of $\sqrt{\Delta x_E^2}$ proportional to t, we will ignore the cross LP-UP oscillating terms where $\beta \neq \alpha$ (note this is consistent with our neglect of oscillations in the previous treatment of P_M). It follows in this case that

$$P_M \Delta x_E^2(t) = -\sum_\alpha \int_{-\infty}^{+\infty} \left[\left(\frac{\partial^2}{\partial q^2}\gamma^*_{\alpha q}\right)\gamma_{\alpha q} - it\left(\frac{\partial}{\partial q}v_g^{\alpha q}\right)|\gamma_{\alpha q}|^2 - 2itv_g^{\alpha q}\left(\frac{\partial}{\partial q}\gamma^*_{\alpha q}\right)\gamma_{\alpha q} - (v_g^{\alpha q}t)^2|\gamma_{\alpha q}|^2\right] dq \tag{51}$$

Note from the above expression that the time-independent contribution gives the exciton spread at $t = 0$

i.e.,

$$P_M \Delta x_E^2(0) = -\sum_\alpha \int_{-\infty}^{+\infty} \left(\frac{\partial^2}{\partial q^2} \gamma^*_{\alpha q} \right) \gamma_{\alpha q} dq. \tag{52}$$

It can be shown with integration by parts that the $\mathcal{O}(t)$ term in Eq. 51 is given by

$$itv_g^{\alpha q} \left(\frac{\partial}{\partial q} \gamma_{\alpha q} \right) \gamma^*_{\alpha q} - itv_g^{\alpha q} \left(\frac{\partial}{\partial q} \gamma^*_{\alpha q} \right) \gamma_{\alpha q} = -2 v_g^{\alpha q} t \times \mathrm{Im} \left[\gamma^*_{\alpha q} \frac{\partial}{\partial q} \gamma_{\alpha q} \right] \tag{53}$$

Hence, it can be seen that if $\gamma_{\alpha q} = c_q \Pi_{\alpha q}$ is real this term vanishes. In the case of Gaussian wave packets in the continuum limit, we have

$$c(q) = \frac{1}{\sqrt{2\pi}} \int_{-\infty}^{+\infty} c(x) e^{-iqx} dx = \frac{1}{2\pi\sigma_x} \int_{-\infty}^{+\infty} e^{-\frac{(x-x_0)^2}{2\sigma_x}} e^{i(\bar{q}_0 - q)x} dx \tag{54}$$

$$\frac{1}{2\pi\sigma_x} \int_{-\infty}^{+\infty} e^{-\frac{(x-x_0)^2}{2\sigma_x}} [\cos{(\bar{q}_0 - q)x} + i \sin{(\bar{q}_0 - q)x}] dx \ . \tag{55}$$

Due to parity considerations, the sine part of this expression vanishes and $c(q)$ must be real. Consequently, Eq. 53 is zero for Gaussian wave packets.

It follows that the ballistic spread velocity v_0 for a Gaussian wave packet is given by

$$v_0^2 = \frac{\Delta x_E^2(t) - \Delta x_E^2(0)}{t^2} = \frac{1}{P_M} \int_{-\infty}^{+\infty} |c(q)|^2 \left[\left(v_g^{\mathrm{LP}}(q) \Pi_{\mathrm{LP}q} \right)^2 + \left(v_g^{\mathrm{UP}}(q) \Pi_{\mathrm{UP}q} \right)^2 \right] dq \ . \tag{56}$$

Defining an effective group velocity as $v_{\alpha q}^{\mathrm{eff}} = v_g^\alpha(q) \Pi_{\alpha q}$ and disregarding the (here constant) prefactor P_M we arrive at the following final estimate for the observed ballistic exciton spread velocity

$$v_0^2 \approx \int_{-\infty}^{+\infty} |c(q)|^2 \left[(v_{\mathrm{LP}q}^{\mathrm{eff}})^2 + (v_{\mathrm{UP}q}^{\mathrm{eff}})^2 \right] dq \ . \tag{57}$$

2 Exciton migration probability

In this section, we obtain insight into the exciton migration probability defined in the main text as

$$\chi(t) = 1 - \frac{1}{P_M(t)} \sum_{n \in \mathcal{I}} |\langle n|\psi(t)\rangle|^2, \tag{58}$$

where $\mathcal{I}$ is the integer interval $[n_{\min}, n_{\max}]$ containing the indices of the dipoles comprising 99% of the initial wave packet probability. For the sake of simplicity, we ignore $P_M(t)$ below since both at very early times $t \to 0$ and late times $t \to \infty$, $P_M(t)$ is approximately constant. Let $P_n(t)$ denote the time-dependent probability to to detect an exciton at the nth site and $\{|A\rangle, |B\rangle, ...\}$ correspond to the set of eigenstates of H with eigenvalues $E_A = \hbar\omega_A$, etc. It follows that we can write the probability $P_n(t)$ to detect an exciton at the nth site at time t is given by

$$\begin{aligned} P_n(t) = |\langle n|\psi(t)\rangle|^2 &= \left| \sum_A \langle n|A\rangle \langle A|\psi_0\rangle e^{-i\omega_A t} \right|^2 \\ &= \left| \sum_A \sum_{m_1} A_n A^*_{m_1} c_{m_1} e^{-i\omega_A t} \right|^2 \\ &= \sum_{A,B} \sum_{m_1,m_2} A_n A^*_{m_1} B^*_n B_{m_2} c_{m_1} c^*_{m_2} e^{-i(\omega_A - \omega_B)t}. \end{aligned} \tag{59}$$

where A_n corresponds to the probability amplitude to detect an exciton at site $|n\rangle$ when the system is in eigenstate $|A\rangle$, and $c_n = \langle n|\psi_0\rangle$ as in the previous section. We can decompose $P_n(t)$ into

$$P_n(t) = P_n + \Delta P_n(t), \tag{60}$$

where P_n corresponds to the time-independent asymptotic part and $\Delta P_n(t)$ is the time-fluctuating contribution to $P_n(t)$. Each of these terms is given explicitly by

$$P_n = \sum_A \sum_{m_1,m_2} |A_n|^2 A^*_{m_1} A_{m_2} c_{m_1} c^*_{m_2}, \tag{61}$$

$$\Delta P_n(t) = \sum_A \sum_{B \neq A} \sum_{m_1,m_2} A_n A^*_{m_1} B^*_n B_{m_2} c_{m_1} c^*_{m_2} e^{-i(\omega_A - \omega_B)t}. \tag{62}$$

Given the definition of $\chi(t)$ in Eq. 58, it follows that we may also define an asymptotic time-independent part contribution χ and an oscillatory contribution $\Delta\chi(t)$ such that $\chi(t) = \chi + \Delta\chi(t)$ given by

$$\chi \approx 1 - \sum_{A} \sum_{n\in\mathcal{I}} \sum_{m_1,m_2} |A_n|^2 A^*_{m_1} A_{m_2} c_{m_1} c^*_{m_2}, \tag{63}$$

$$\Delta\chi(t) \approx -\sum_{A} \sum_{B\neq A} \sum_{n\in\mathcal{I}} \sum_{m_1,m_2} A_n A^*_{m_1} B^*_n B_{m_2} c_{m_1} c^*_{m_2} e^{-i(\omega_A-\omega_B)t}, \tag{64}$$

where the approximate character of the identities emphasizes our neglect of $P_M(t)$ in the definition of $\chi(t)$. We examine χ and $\Delta\chi(t)$ next starting with the time-independent term which we rewrite as

$$\begin{aligned}\chi = 1 &- \sum_{A} \sum_{n\in\mathcal{I}} |A_n|^4 |c_n|^2 - \sum_{A} \sum_{n\in\mathcal{I}} \sum_{m\neq n} |A_n|^2 |A_m|^2 |c_m|^2 \\ &- \sum_{A} \sum_{n\in\mathcal{I}} \sum_{m\neq n} |A_n|^2 \left(A^*_n A_m c_n c^*_m + A^*_m A_n c_m c^*_n\right) - \sum_{A} \sum_{n\in\mathcal{I}} \sum_{m_1\neq n} \sum_{m_2\neq m_1,n} |A_n|^2 A^*_{m_1} A_{m_2} c_{m_1} c^*_{m_2}.\end{aligned} \tag{65}$$

The fluctuating term can be written likewise as

$$\begin{aligned}\Delta\chi(t) = &- \sum_{A} \sum_{B\neq A} \sum_{n\in\mathcal{I}} |A_n|^2 |B_n|^2 |c_n|^2 \cos[(\omega_A - \omega_B)t] \\ &- \sum_{A} \sum_{B\neq A} \sum_{n\in\mathcal{I}} \sum_{m\neq n} \left(A_n A^*_m B^*_n B_m |c_m|^2 + |A_n|^2 B^*_n B_m c_n c^*_m + A_n A^*_m |B_n|^2 c_m c^*_n\right) e^{-i(\omega_A-\omega_B)t} \\ &- \sum_{A} \sum_{B\neq A} \sum_{n\in\mathcal{I}} \sum_{m_1\neq n} \sum_{m_2\neq m_1,n} A_n A^*_{m_1} B^*_n B_{m_2} c_{m_1} c^*_{m_2} e^{-i(\omega_A-\omega_B)t}.\end{aligned} \tag{66}$$

Note that

$$\frac{\mathrm{d}}{\mathrm{d}t}\chi(t) = \frac{\mathrm{d}}{\mathrm{d}t}\Delta\chi(t), \tag{67}$$

and $\mathrm{d}\Delta\chi(t)/\mathrm{d}t \to 0$ as $t \to 0^+$. Therefore, the early growth of $\chi(t)$ is characterized by

$$G = \lim_{t\to 0^+} \frac{1}{2} \frac{\mathrm{d}^2}{\mathrm{d}t^2} \Delta\chi(t). \tag{68}$$

Strong disorder limit. To simplify, we perform the disorder average of χ which we denote by $\overline{\chi}$ (our notation for disorder average of a quantity f in this section is given by $\overline{f}$). Under sufficiently strong disorder ($\sigma_M \gg \Omega_R$), we expect $\overline{A^*_{m_1} A_{m_2}} \approx |\overline{A_{m_1}}|^2 \delta_{m_1,m_2}$ for any eigenmode A, and therefore the disorder-averaged

exciton migration probability components satisfy

$$\overline{\chi} \approx 1 - \sum_{A} \sum_{n\in\mathcal{I}} \overline{|A_n|^4} |c_n|^2 - \sum_{A} \sum_{n\in\mathcal{I}} \sum_{m\neq n} \overline{|A_n|^2}\ \overline{|A_m|^2} |c_m|^2, \tag{69}$$

$$\overline{\Delta\chi(t)} \approx - \sum_{A} \sum_{B\neq A} \sum_{n\in\mathcal{I}} \overline{|A_n|^2 |B_n|^2} |c_n|^2 \cos\left[(\omega_A - \omega_B)t\right]. \tag{70}$$

From the last equation, we directly quantify the early growth of the migration probability from

$$\overline{G} \approx \frac{1}{2} \sum_{A} \sum_{B\neq A} \sum_{n\in\mathcal{I}} \overline{|A_n|^2 |B_n|^2} |c_n|^2 (\omega_A - \omega_B)^2. \tag{71}$$

Weak disorder limit. In the weak disorder case,

$$G = \frac{1}{2} \sum_{A} \sum_{B\neq A} \sum_{n\in\mathcal{I}} \sum_{m_1,m_2} A_n A^*_{m_1} B^*_n B_{m_2} c_{m_1} c^*_{m_2} (\omega_A - \omega_B)^2 \tag{72}$$

Assuming only modes with $q\sigma_x \ll 1$ contribute significantly, we can take the long wavelength limit and ignore the phase difference of eigenmode amplitudes in distinct sites, thus considerably simplifying G

$$G \approx \frac{1}{2N_{\mathcal{I}}} \sum_{A} \sum_{B\neq A} \sum_{n\in\mathcal{I}} \sum_{m_1,m_2} |A_n|^2 |B_n|^2 (\omega_A - \omega_B)^2, \tag{73}$$

where we made the replacement $c_{m_1} c^*_{m_2} \approx 1/N_{\mathcal{I}}$ and $N_{\mathcal{I}}$ is the number of elements of $\mathcal{I}$.

3 Wave packets at long propagation times

Fig. S1 shows the shape of wave packets at 1, 2, and 3 ps. We observe that, except when $\sigma_M/\Omega_R = 5\%$, the wave packet remains localized around its initial region. Therefore, the asymptotic behavior seen in Figure 3 (main text) can be attributed to Anderson localization of the excitonic wave packet.

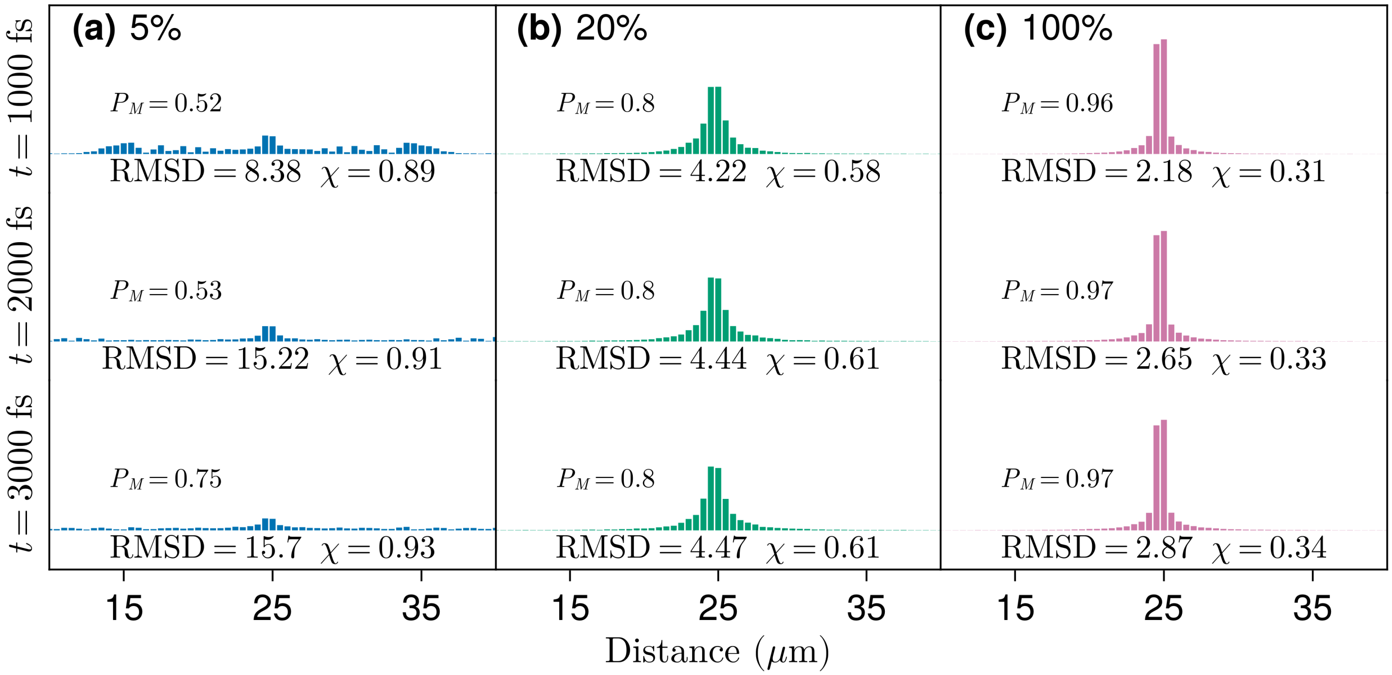

Figure S1: Average exciton wave packet profiles at different time delays and relative disorder strength (σ_M/Ω_R) of 5%, 20%, and 100% for (a), (b) and (c), respectively. In all cases, $\Omega_R = 0.1$ eV and the wave packet initial width and mean quasimomentum are $\sigma_x = 120$ nm and $\bar{q}_0 = 0$. Probabilities are grouped in bins containing 50 dipoles spanning 0.5 μm. P_M, RMSD, and χ are defined in Equations (74),(38), and (76), respectively. Each profile is obtained from the average of 100 disorder realizations.

Fig. S2 shows the probability of finding an excitation on a site near the edges of the quantum wire. Since we have employed periodic boundary conditions, the exciton wave packet will go around the wire (which can be thought of more precisely as a ring) and start interfering with itself, creating finite-size artifacts. Moreover, the computation of RMSD also becomes ambiguous once the wave packet travels around. Hence, to guarantee that our results are not plagued with these artifacts, we monitor the probability of detecting the exciton at the most distant sites with respect to the initial excitation spot. From Fig. S2, we see that this probability remains small under enough disorder ($\sigma_M/\Omega_R \geq 0.2$). In the femtosecond window, even the small disorder simulation ($\sigma_M/\Omega_R = 0.05$) has a small excitation probability at the edges. These results are important to ensure that the RMSD asymptote seen in Fig. 3b (main text) is truly disorder-induced localization and not finite-size effects.

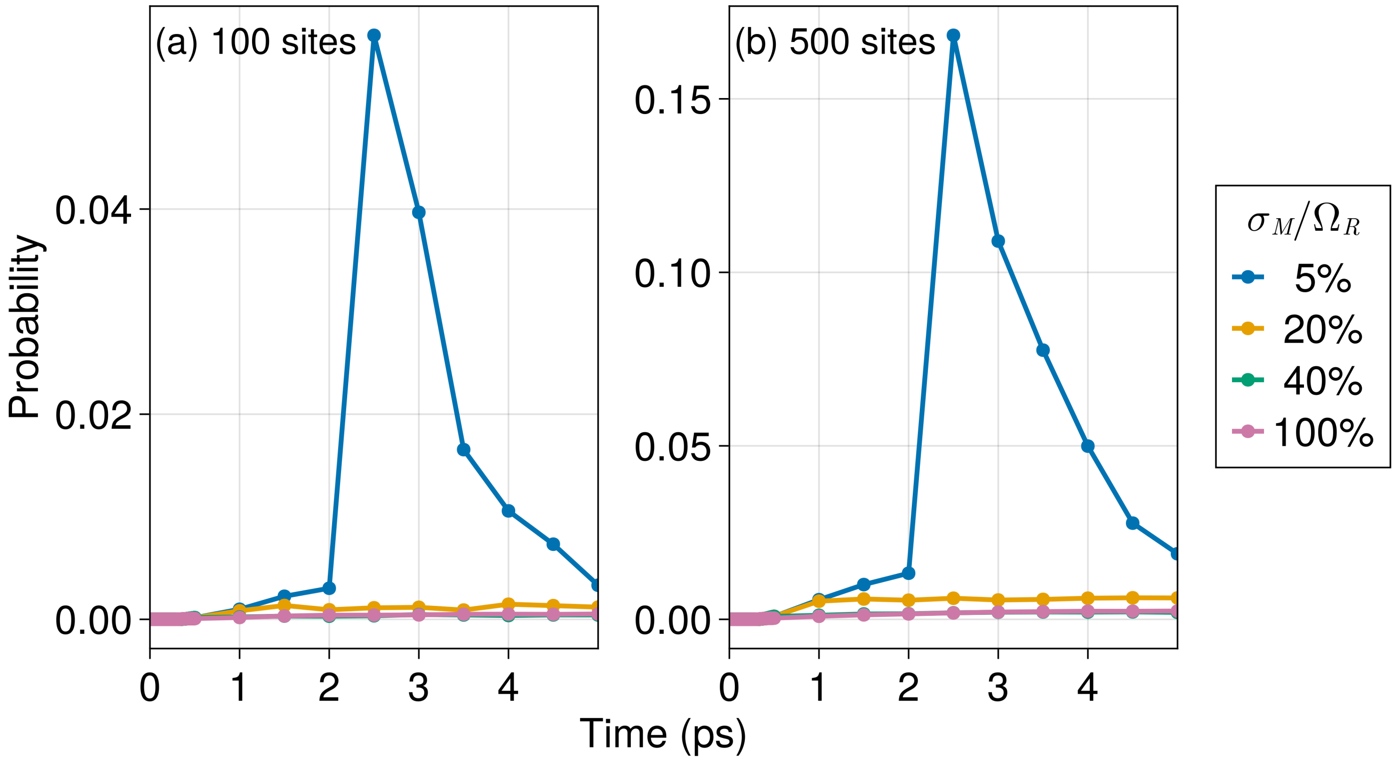

Figure S2: Probability of finding the exciton near the boundary of the wire. This value is computed by summing the excitation probability of **(a)** 100 and **(b)** 500 sites closest to the region of the wire where periodic boundary conditions are enforced ($x = 0$ and $x = 50$ μm). In all cases, $\Omega_R = 0.1$ eV and the wave packet initial width and mean quasimomentum are $\sigma_x = 120$ nm and $\bar{q}_0 = 0$. Each data point is obtained from the average of 100 disorder realizations.

4 Wave packets under DET

In Figs. S3, S4, and S5 we show snapshots of the exciton wave packet at different time steps where we can see the different in the wave packet shape under disorder enhanced transport (DET). In Figs. S3-S5(a) we see that under very strong disorder ($\sigma_M/\Omega_R > 1$) the wave packet decays more quickly in space, but has a more delocalized tail. This decolalization is captured in the RMSD but not in the migration probability (χ) as seen in S3-S5(b).

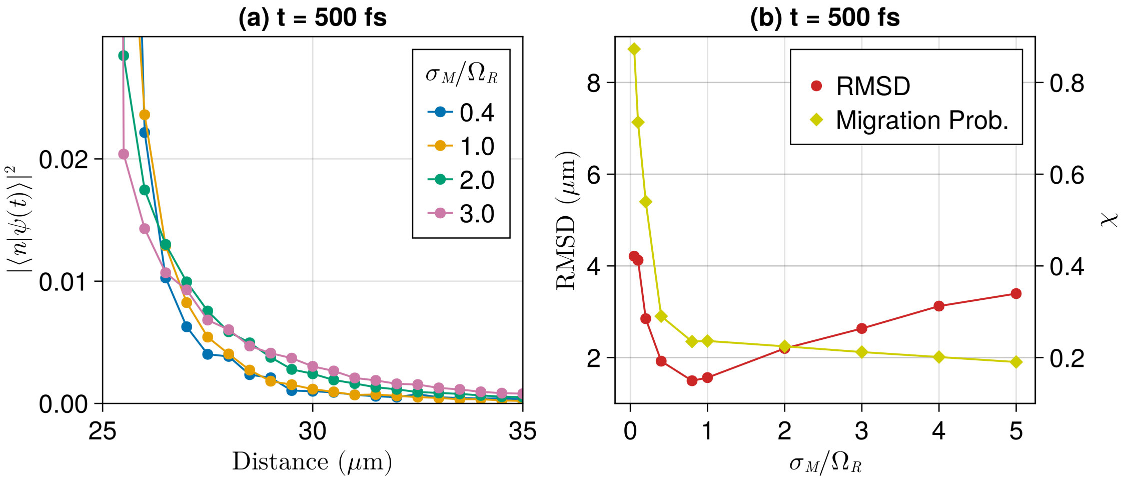

Figure S3: Wave packet properties at $t = 500$ fs: **(a)** Decay as a function of distance at different relative disorder strengths. **(b)** RMSD and migration probabilities at a fixed time step at different disorder strengths. In all cases, $\Omega_R = 0.1$ eV and $\sigma_x = 120$ nm. Other parameters are fixed as in the main text.

'

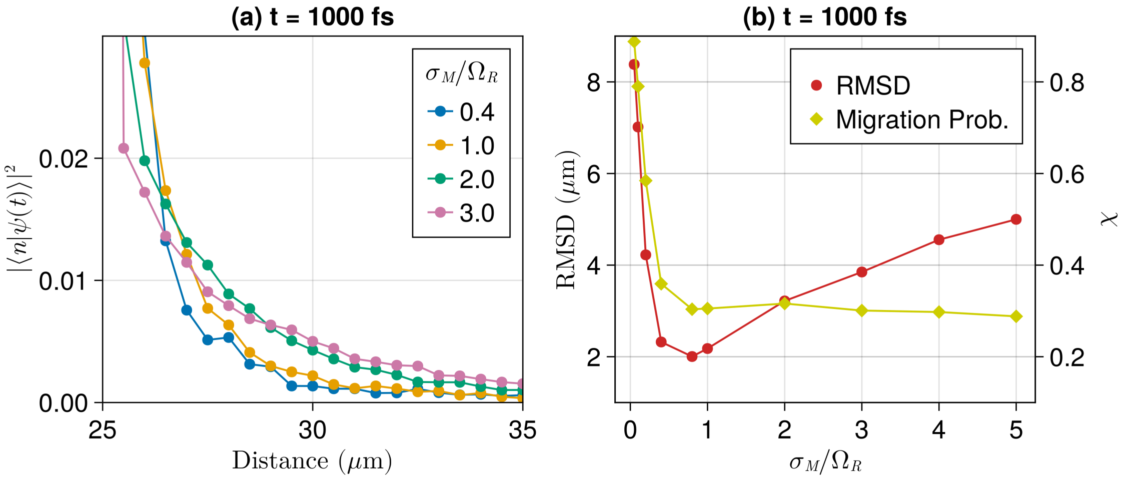

Figure S4: Wave packet properties at $t = 1000$ fs: **(a)** Decay as a function of distance at different relative disorder strengths. **(b)** RMSD and migration probabilities at a fixed time step at different disorder strengths. In all cases, $\Omega_R = 0.1$ eV and $\sigma_x = 120$ nm. Other parameters are fixed as in the main text.

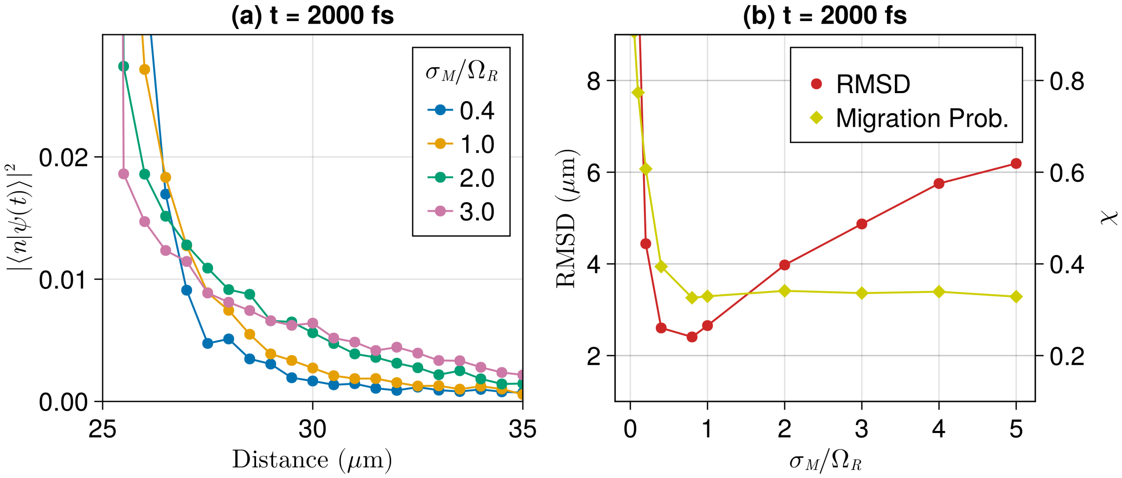

Figure S5: Wave packet properties at $t = 2000$ fs: **(a)** Decay as a function of distance at different relative disorder strengths. **(b)** RMSD and migration probabilities at a fixed time step at different disorder strengths. In all cases, $\Omega_R = 0.1$ eV and $\sigma_x = 120$ nm. Other parameters are fixed as in the main text.

5 Propagation Profiles

Figs. S6-S9 shows the time evolution of the RMSD and migration probability (χ) defined in the main text and repeated below for reference

$$\mathrm{RMSD}(t) = \left[\frac{1}{P_{\mathrm{M}}(t)} \sum_{n}^{N_M} |\langle n; 0|\psi(t)\rangle|^2 (x_n - x_0)^2\right]^{1/2}, \tag{74}$$

$$P_{\mathrm{M}}(t) = \sum_{n}^{N_M} |\langle n; 0|\psi(t)\rangle|^2 . \tag{75}$$

$$\chi(t) = 1 - \frac{1}{P_{\mathrm{M}}(t)} \sum_{n=n_{\mathrm{min}}}^{n_{\mathrm{max}}} |\langle n; 0|\psi(t)\rangle|^2 . \tag{76}$$

It can be seen that the time to reach a steady state (constant RMSD) decreases as the Rabi splitting is increased.

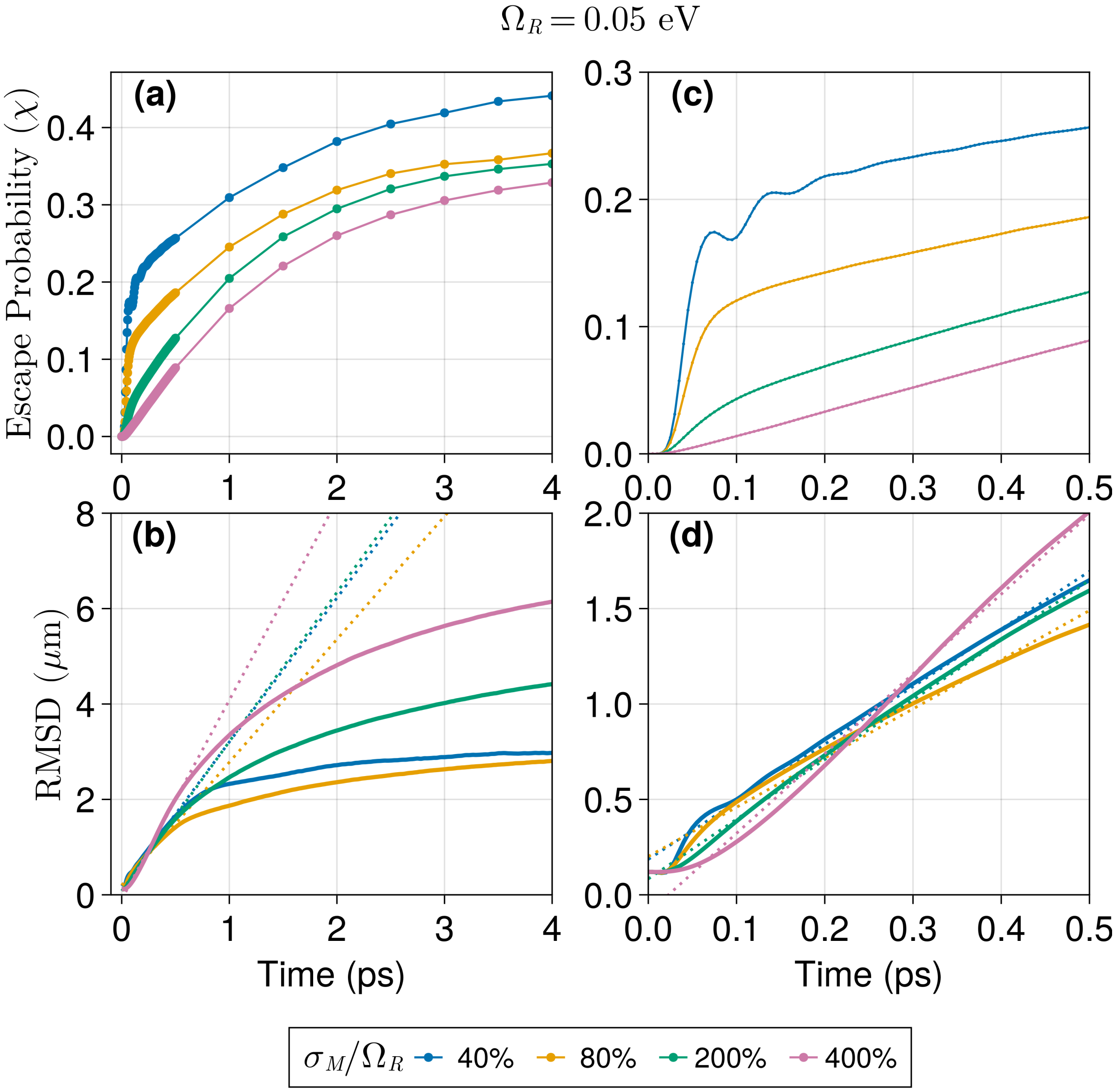

Figure S6: Propagation of exciton wave packets at different disorder strengths measured by **(a)** migration probability (Eq. 76) and **(b)** RMSD (Eq. 74). In all cases, $\sigma_x = 120$ nm and $\Omega_R = 0.05$ eV. Results are averages of 100 disordered realizations. The dotted lines in **(b)** are linear fits on the early propagation ($\leq$ 500 fs) from which slopes are used to measure the initial ballistic velocity (v_0). Panels **(c)** and **(d)** are zoomed in versions of **(a)** and **(b)**, respectively.

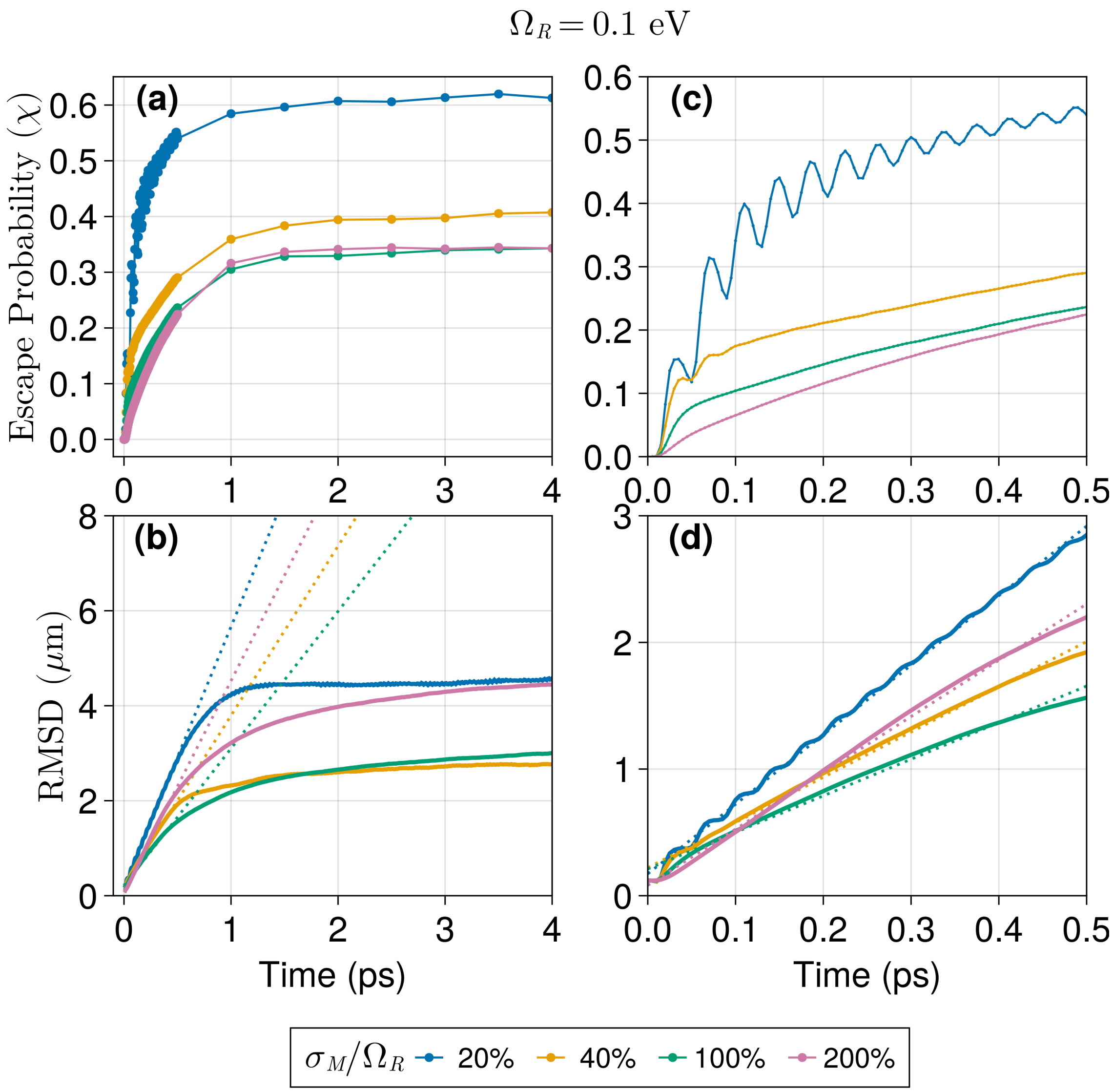

Figure S7: Propagation of exciton wave packets at different disorder strengths measured by **(a)** migration probability (Eq. 76) and **(b)** RMSD (Eq. 74). In all cases, $\sigma_x = 120$ nm and $\Omega_R = 0.1$ eV. Results are averages of 100 disordered realizations. The dotted lines in **(b)** are linear fits on the early propagation ($\leq$ 500 fs) from which slopes are used to measure the initial ballistic velocity (v_0). Panels **(c)** and **(d)** are zoomed in versions of **(a)** and **(b)**, respectively.

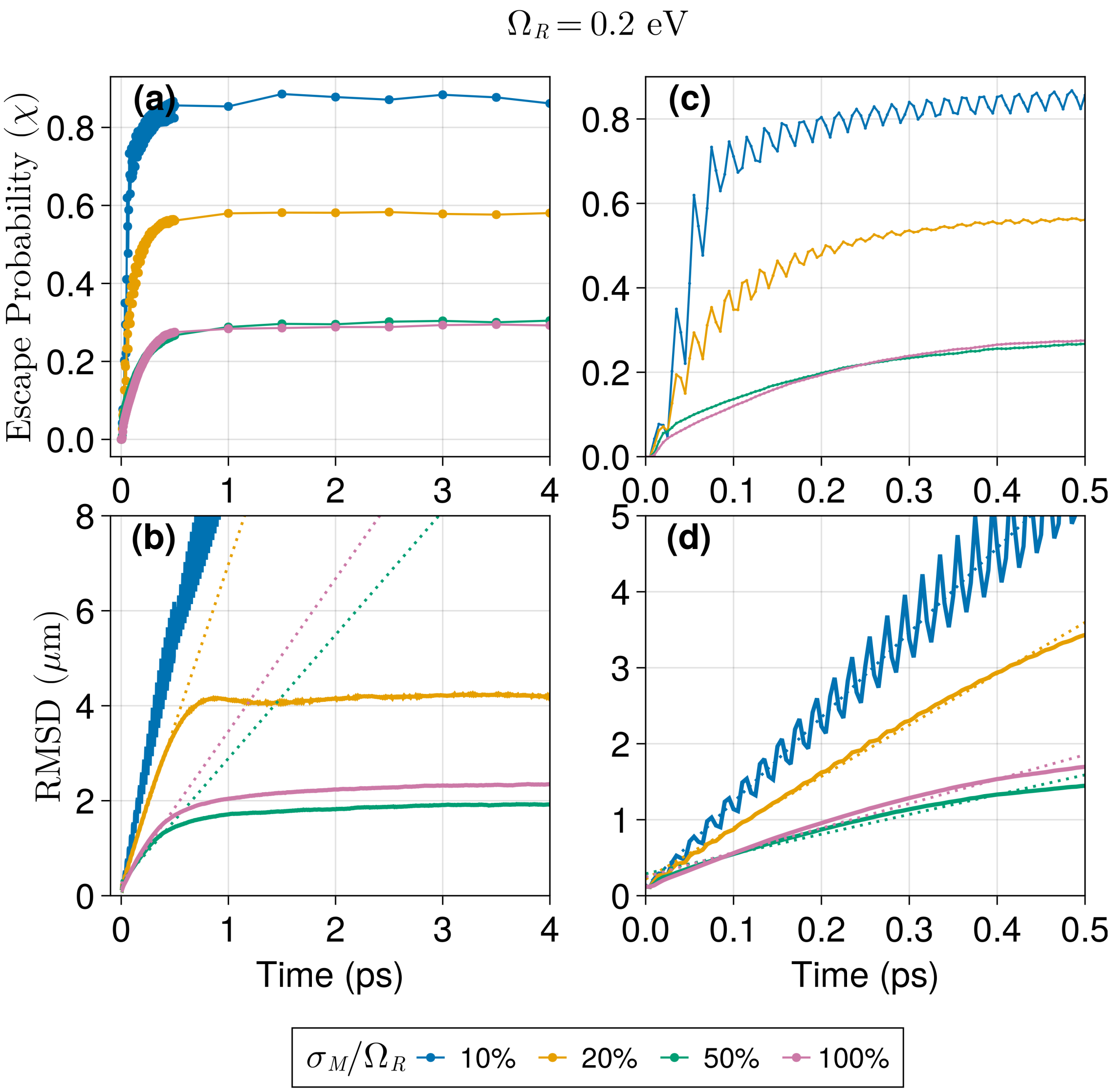

Figure S8: Propagation of exciton wave packets at different disorder strengths measured by **(a)** migration probability (Eq. 76) and **(b)** RMSD (Eq. 74). In all cases, $\sigma_x = 120$ nm and $\Omega_R = 0.2$ eV. Results are averages of 100 disordered realizations. The dotted lines in **(b)** are linear fits on the early propagation ($\leq$ 500 fs) from which slopes are used to measure the initial ballistic velocity (v_0). Panels **(c)** and **(d)** are zoomed in versions of **(a)** and **(b)**, respectively.

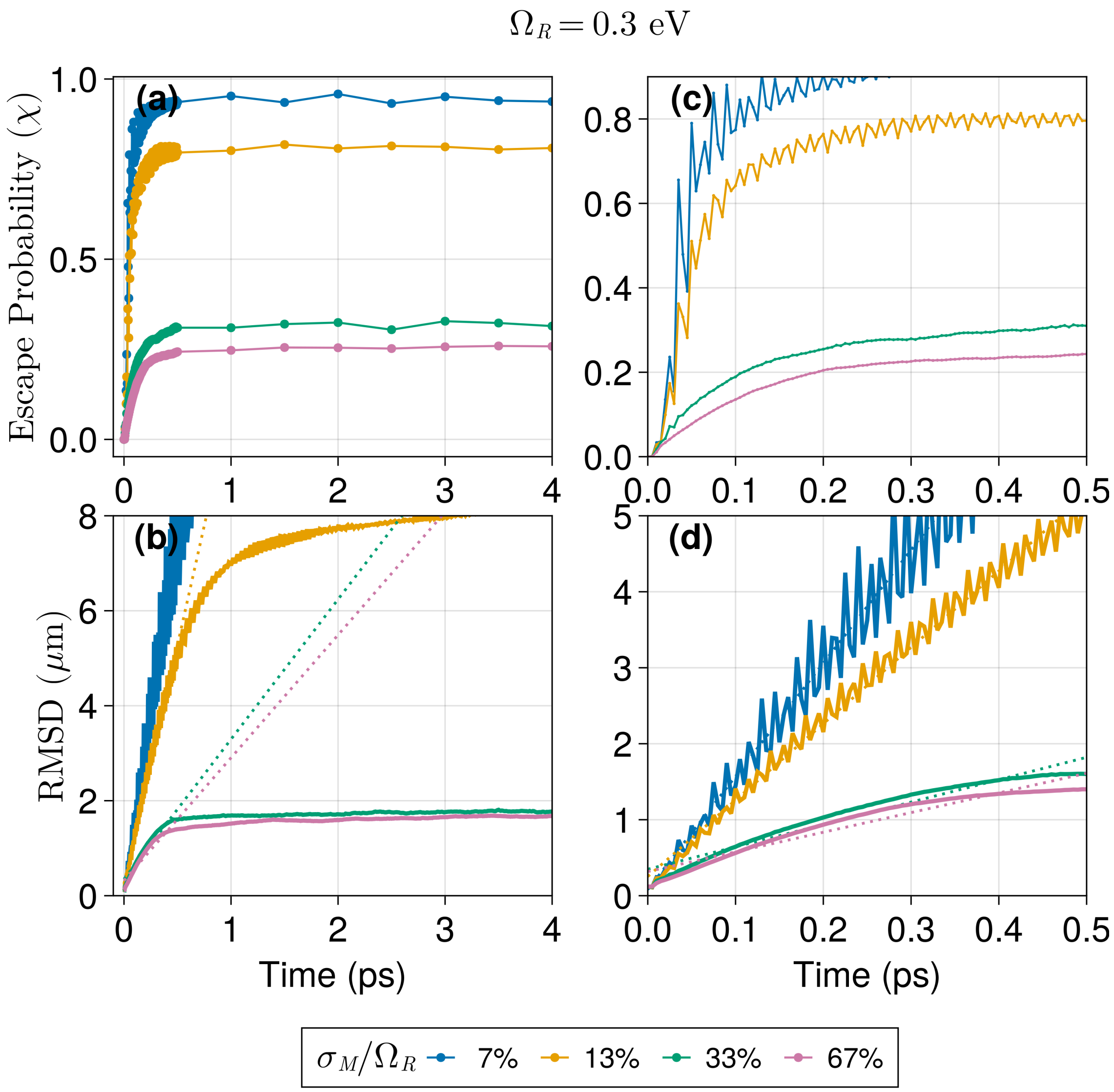

Figure S9: Propagation of exciton wave packets at different disorder strengths measured by **(a)** migration probability (Eq. 76) and **(b)** RMSD (Eq. 74). In all cases, $\sigma_x = 120$ nm and $\Omega_R = 0.3$ eV. Results are averages of 100 disordered realizations. The dotted lines in **(b)** are linear fits on the early propagation ($\leq$ 500 fs) from which slopes are used to measure the initial ballistic velocity (v_0). Panels **(c)** and **(d)** are zoomed in versions of **(a)** and **(b)**, respectively.

6 Linear Fit Range

In the main text, we have used the first 500 fs of our simulations to compute the ballistic velocity (v_0) using a linear fit. Our criterion to choose an interval for a linear fit was 1) long enough periods such that complicated initial features are averaged out, and 2) short enough to prevent the consideration of time steps where Anderson localization starts to appear (around 1 ps). Figs. S10, S11, and S12 show analogs of Figure 4 (main text) using different time intervals for the linear fit (0.1, 0.3, and 0.7 ps, respectively). The choice of time interval is not strictly fundamental for a qualitative analysis as we see an overall agreement for all time intervals. Importantly, The σ_x dependence discussed in the text is maintained in all cases.

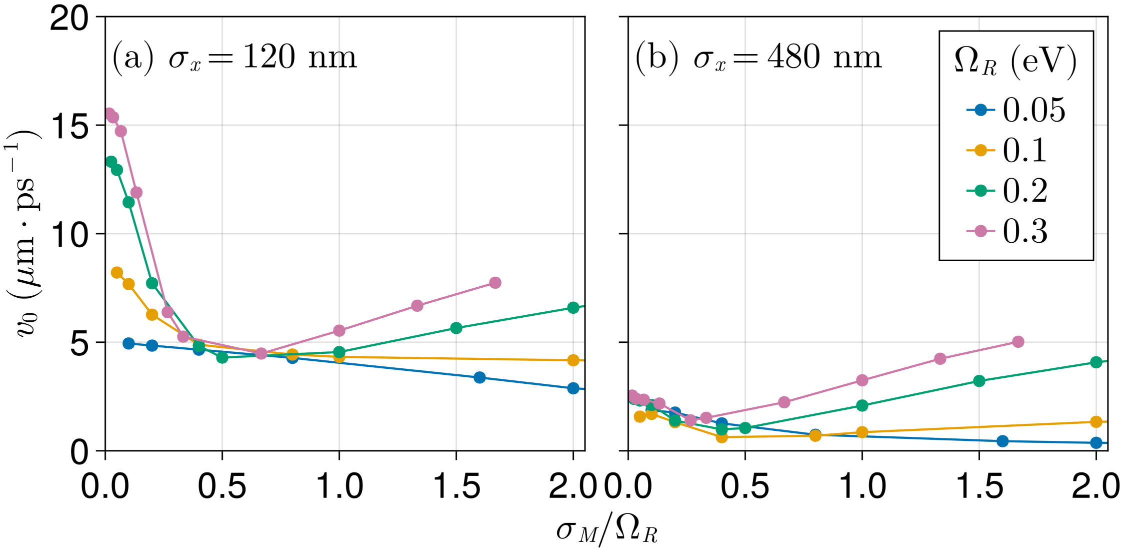

Figure S10: Initial spread velocity (v_0) for various systems at different relative disorder strength and Rabi splitting values for wave packets with a **(a)** narrow and **b** broad initial spread values (σ_x). Each point is the average of 100 disordered realizations. v_0 was computed as the slope of a linear fit of RMSD values in the initial 100 fs of simulation (see Figure 3) in the main text.

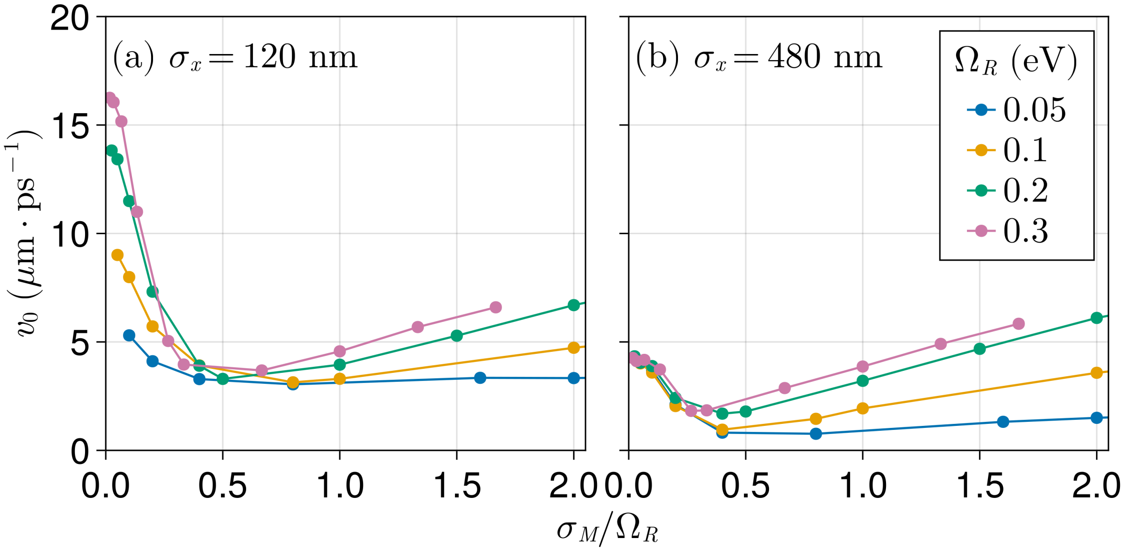

Figure S11: Initial spread velocity (v_0) for various systems at different relative disorder strength and Rabi splitting values for wave packets with a **(a)** narrow and **b** broad initial spread values (σ_x). Each point is the average of 100 disordered realizations. v_0 was computed as the slope of a linear fit of RMSD values in the initial 300 fs of simulation (see Figure 3) in the main text.

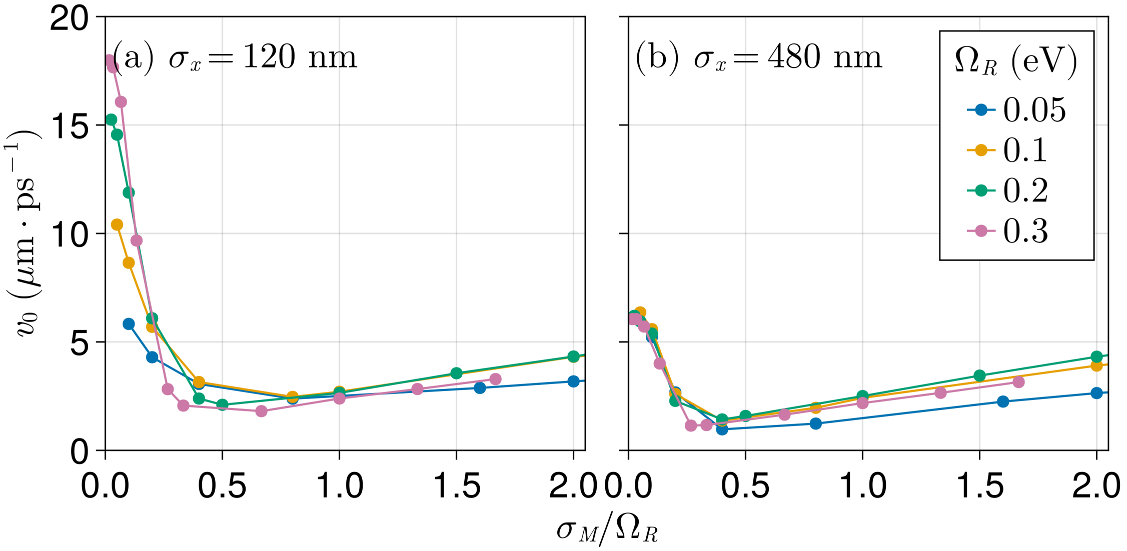

Figure S12: Initial spread velocity (v_0) for various systems at different relative disorder strength and Rabi splitting values for wave packets with a **(a)** narrow and **b** broad initial spread values (σ_x). Each point is the average of 100 disordered realizations. v_0 was computed as the slope of a linear fit of RMSD values in the initial 700 fs of simulation (see Figure 3) in the main text.

7 v_0 for detuned cavities

Figure S13 shows v_0 (see Figure 3b on the main text for more details about how v_0 is obtained) as a function of detuning for several initial excitons spread values (σ_x). In particular, the left column panels (a, c, e, g) display results using a zero average initial momentum ($\bar{q}_0 = 0$) while the right column (b, d, f, h) shows results for a non-zero quasi-momentum ($\bar{q}_0 = 0.008$ nm^{-1}). Results presented in this figure numerically verify the predictions from the effective group velocity model (see Figure 8 in the main text). Namely, we see that when $\bar{q}_0 = 0$:

1. Narrow wave packets have higher mobility.

2. Blueshifted cavities have always slower excitons than resonant ones.

3. Redshifted cavities have more mobility excitons if the wave packets are narrow enough.

For $\bar{q}_0 = 0.008$ nm^{-1} (note that this is near the $v_{\alpha q}^{\text{eff}}$ peak for the redshifted cavity in Figure 8), we verify the following predictions:

1. Redshifted cavities have faster excitons than resonant ones.

2. Broader wave packets are more mobile than narrow ones.

3. Blueshifted cavities have always slower excitons than resonant ones.

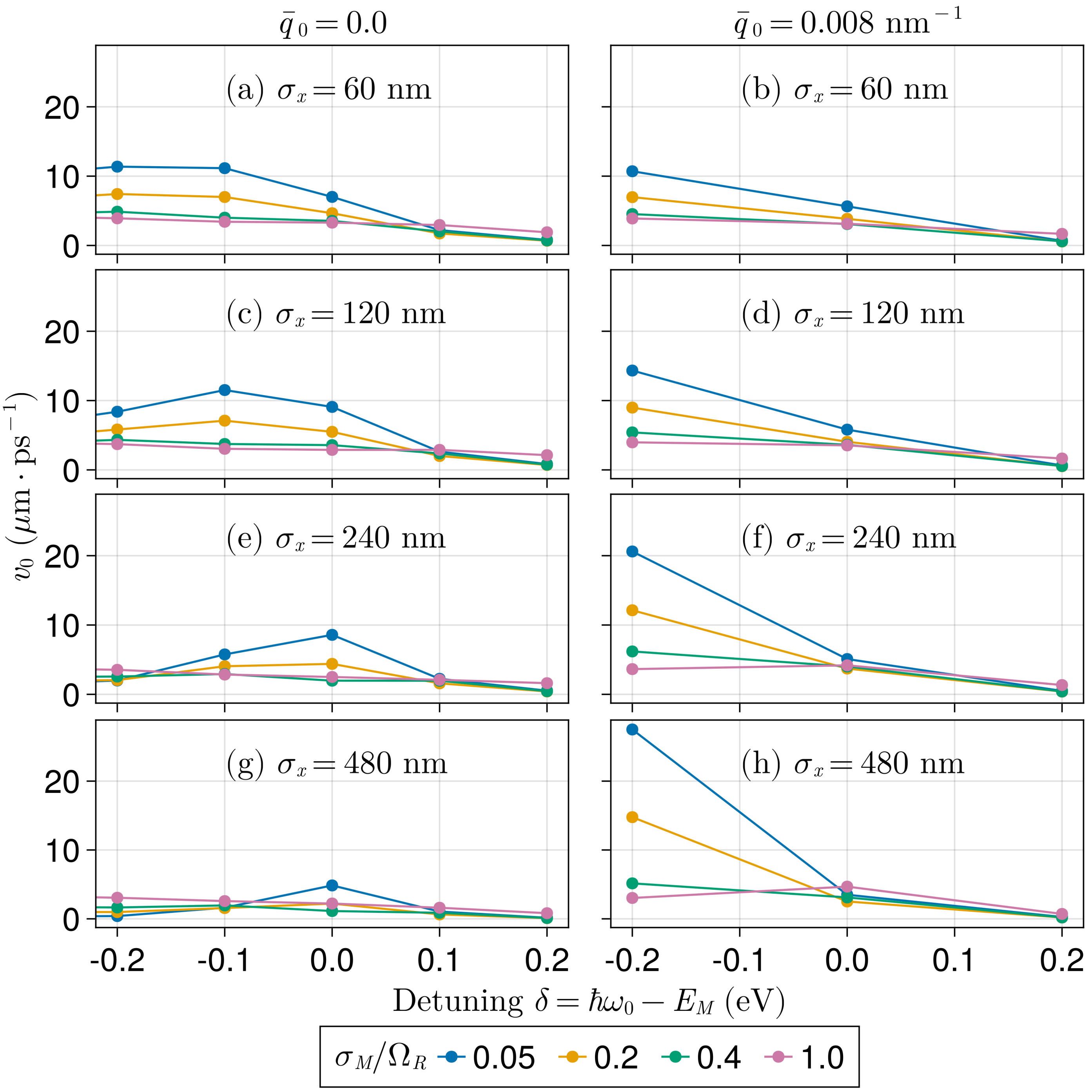

Figure S13: Ballistic velocity (v_0) as a function of the cavity detuning (δ) for several disorder strengths (σ_M/Ω_R). Left column panels (a, c, e, g) show results for excitons with zero quasi-momentum ($\bar{q}_0 = 0$) whereas in the right column panels excitons have $\bar{q}_0 = 0.008\ \mathrm{nm}^{-1}$. In each panel, a different initial spread is used (σ_x).

Figure S14 shows how v_0 and the maximum RMSD depend on the cavity detuning. This is similar to Figure 7 of the main text, but with more redshifted values to explore whether an extremum exists in this direction. While we do not observe a peak, we see that both v_0 and the maximum RMSD reach an asymptotic behavior, i.e. further changes in the cavity detuning (towards negative values) do not change the overall exciton mobility. We highlight that, for very redshifted cavities, the description of the photonic degrees of freedom becomes poorer as higher energy bands (n_y, $n_z > 1$) become more relevant. Therefore, these results represent the theoretical limit in a single-band cavity system.

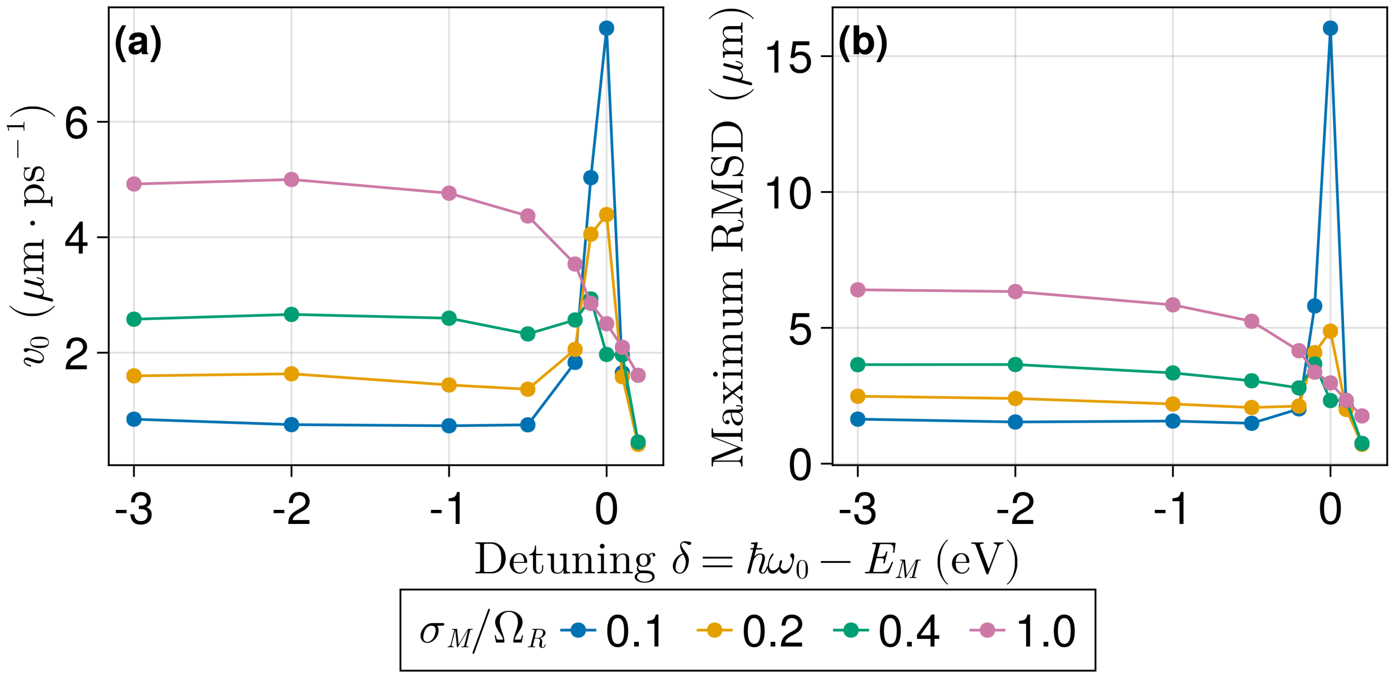

Figure S14: Disorder-dependent detuning effects on coherent exciton transport measured by **(a)** the ballistic velocity (v_0), and **(b)** the maximum RMSD over 5 ps. In all cases σ_x = 240 nm and Ω_R = 0.1 eV. Each data point corresponds to an average over 100 disorder realizations.

8 Strong Coupling Signatures

Figure S15 shows mean energy (and fluctuations) vs maximum value of photonic q for the eigenstates with more than 10% photonic content obtained from 25 realizations of a system of 1000 dipoles coupled to 401 photonic modes. As expected, at small and moderate disorder ($\sigma_M/\Omega_R \leq 0.4$), two distinct curves separated by approximately Ω_R are observed, corresponding to LP (yellow) and UP (blue) branches of the quasiparticles emergent from strong light-matter coupling. Figure S15 also shows that further increase of disorder shrinks the gap between these two branches, and when the relative disorder σ_M/Ω_R approaches 1, it is no longer possible in general to discern the two branches at the resonance wave vector ($q = 0$). That is, the characteristic splitting associated with the strong coupling regime is no longer observable. We ascribe no meaning to the irregularities and breakdown of inversion symmetry at large and moderate disorder, as these features likely reflect the smallness of the system size and the limited number of disorder realizations we employed to identify the breakdown of strong coupling (as defined by the gap between LP and UP at the resonant wave vector $q = 0$).

Figure S16 presents the change in the exciton content of the wave packet over time where Rabi oscillations can be observed. Under weak disorder **(a)** clear oscillations with a frequency of approximately 41 fs^{-1} (which corresponds to the Rabi splitting of 0.1 eV used here) can be verified. Under stronger values of disorder, shown in Figure S16b, we see that Rabi oscillations are strongly suppressed. When σ_M/Ω_R is close to one, is no longer possible to identify any oscillation.

The results presented in Figures S15 and S16 are typically employed in criteria for the strong coupling regime. Despite the absence of dissipation in our model, we see that strong static disorder is capable of removing the typical signatures of the strong coupling regime. Hence, one may consider that when the static disorder is comparable to the Rabi splitting (that is, the expected collective coupling value) a different regime emerges. Nonetheless, our model still predicts coherent transport, therefore we consider it to be distinct from the weak coupling situation where non-interacting molecules would not exchange energy.

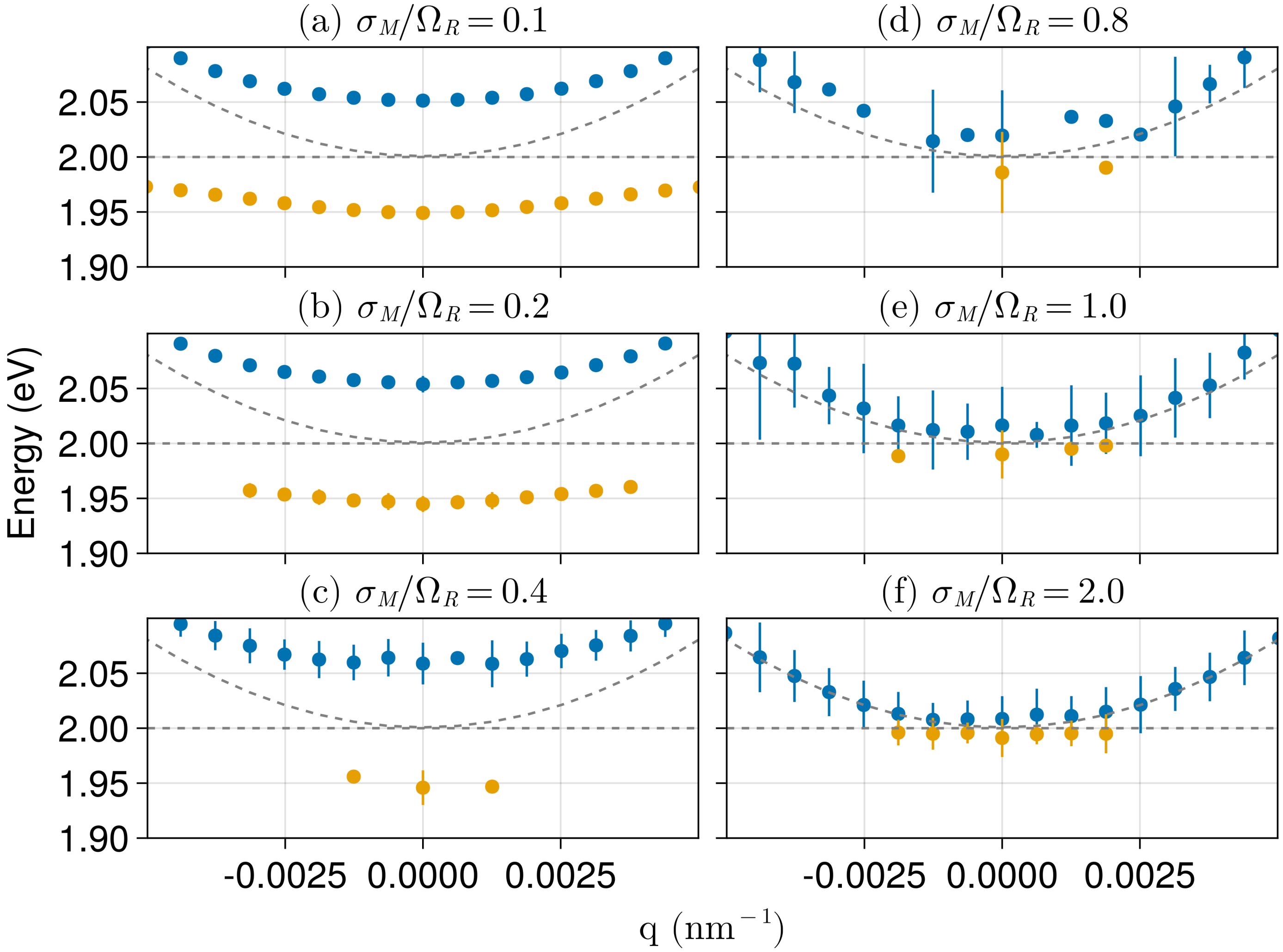

Figure S15: Average energy vs photon q with maximum probability for bright modes (total photon content $> 10\%$) of systems under increasing disorder. Each point represent the mean energy obtained from the eigenstates with more than 10% photonic content in 25 realizations of the light-matter Hamiltonian with the specified σ_M/Ω_R and Ω_R fixed at 0.1 eV. For the sake of simplicity, we show results for systems containing $N_M = 1000$ molecules and 401 photonic modes. The graphs show the gradual closing of the polariton gap (here represented by $E_{\mathrm{UP},q} - E_{\mathrm{LP},q}$ with $q = 0$) as σ_M/Ω_R increases. Finer features (e.g., the number of points in the LP and UP belonging to specific intervals of q) are unlikely to be converged due to the small system size and the number of realizations we employed. However, this lack of convergence is irrelevant to our analysis of the polariton gap.

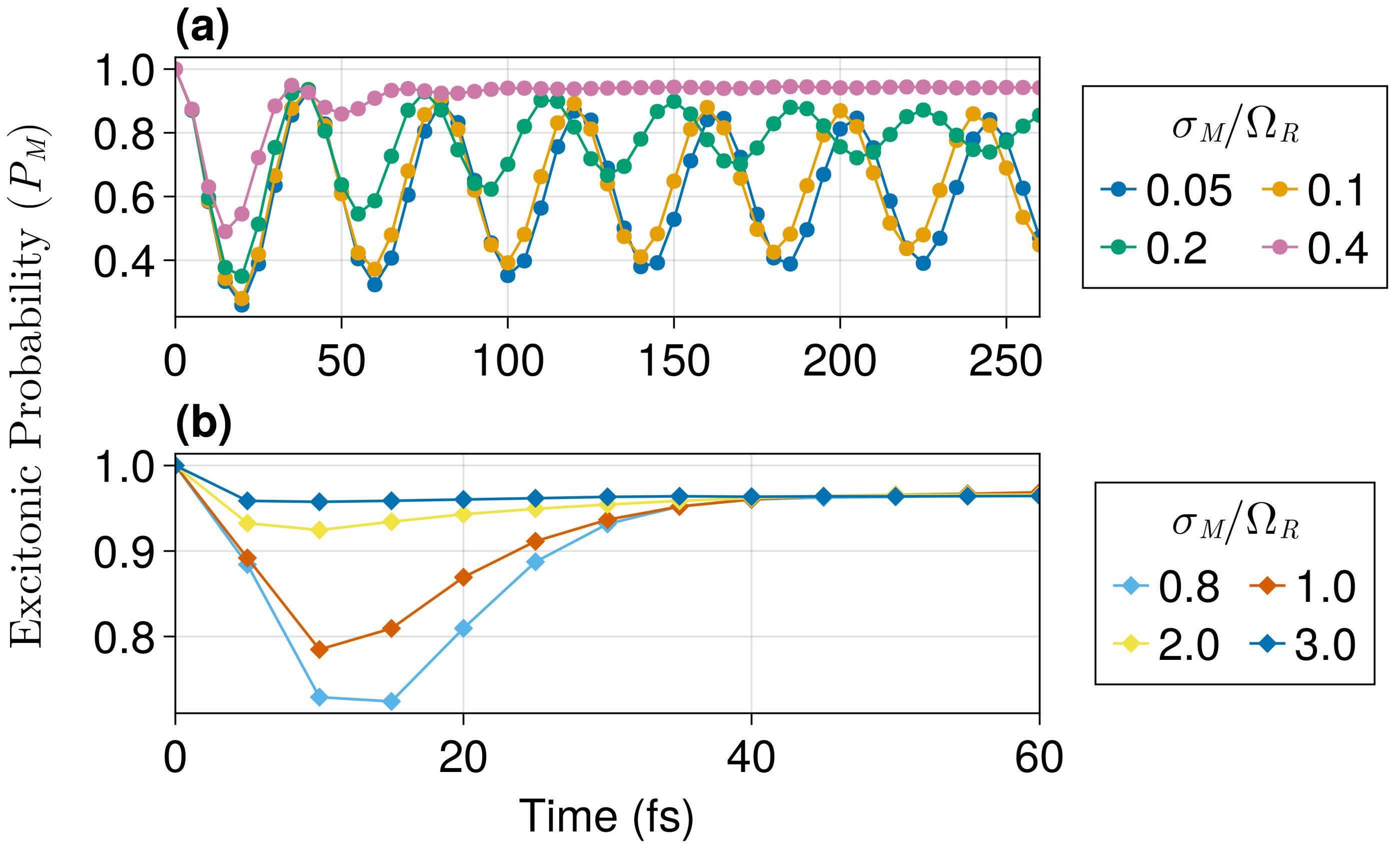

Figure S16: Demonstration of the effect of disorder on Rabi oscillations. The excitonic probability is the summed probability that any site is in its excited state. In all cases the Rabi splitting is fixed at 0.1 eV and each point is the average of 100 realizations.
